# Lanthanide-doped nanocrystals enable organic room-temperature phosphorescence in solution through direct triplet excitation

**DOI:** 10.1038/s41557-026-02159-w

**Published:** 2026-05-25

**Authors:** Huangtianzhi Zhu, Rakesh Arul, Zhao Jiang, Bofeng Xue, Matteo Fornasarig, Danillo Valverde, Lars van Turnhout, Yunzhou Deng, Alasdair Tew, Yoann Olivier, David Beljonne, Zhongzheng Yu, Akshay Rao

**Affiliations:** 1 Department of Chemistry, Zhejiang University, Hangzhou, China; 2 Cavendish Laboratory, University of Cambridge, Cambridge, UK; 3 Laboratory for Computational Modelling of Functional Materials, Namur Institute of Structured Matter, University of Namur, Namur, Belgium; 4 Laboratory for Chemistry of Novel Materials, University of Mons, Mons, Belgium

**Keywords:** Organic-inorganic nanostructures, Excited states

## Abstract

Organic triplet excitons are of great interest for applications in optoelectronics, photochemistry and theranostics. Due to spin-selection rules, triplets are ‘dark states’, rendering direct photoexcitation from the ground state and efficient phosphorescent emission nearly impossible. Overcoming these spin-dependent limitations is a long-standing challenge. Here we report a universal method to brighten organic triplet excitons by attaching chromophores onto the surface of lanthanide-doped nanocrystals, enabling spin-exchange coupling between the unpaired spins of lanthanide ions and organic molecules. This allows direct photoexcitation of the organic triplets, and room-temperature, nanosecond-timescale, oxygen-insensitive phosphorescence in both solution and film under ambient conditions. Different organic chromophores and lanthanide ions are combined to obtain phosphorescence in the visible and near-infrared range. Compared with common organic phosphorescence, which only exists in crystals or at low temperature, the triplet emission established here does not require crystallization, low temperatures or an inert atmosphere. Our approach could open avenues to the application of room-temperature organic phosphorescence in optoelectronic devices and biological labelling and imaging.

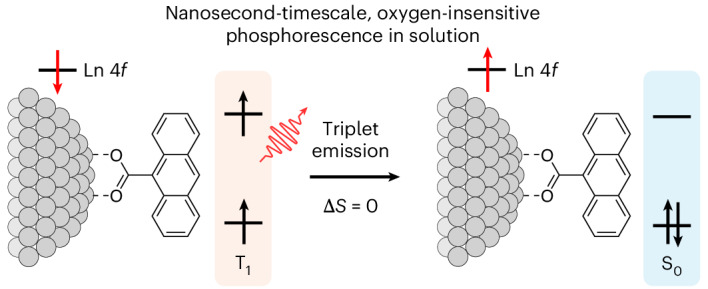

## Abstract (web summary)

Organic triplet excitons are ‘dark states’, rendering direct photoexcitation from the ground state and efficient phosphorescence difficult. Now it has been shown that attaching chromophores to lanthanide-doped nanocrystals enables spin-exchange-mediated direct triplet excitation and nanosecond-timescale, oxygen-insensitive phosphorescence in solution and under ambient conditions.

## Main

**Fig. 1:**
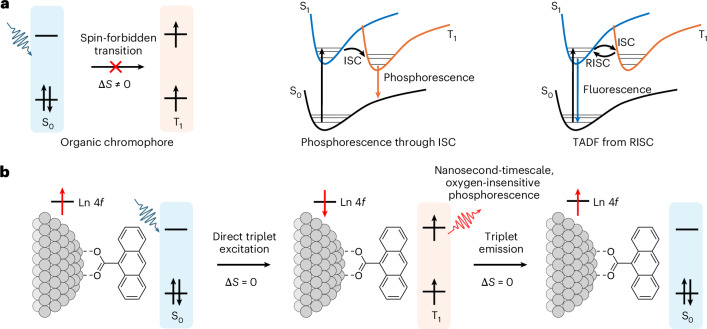
Comparison between conventional ISC-mediated triplet generation and spin-exchange-promoted direct triplet excitation and emission. **a**, Schematic illustration (left) of the spin-forbidden S_0_ → T_1_ transition in an isolated organic chromophore. Δ*S* represents the change of the spin quantum number. To harvest the energy of triplet excitons, for example, via phosphorescence (middle) and TADF (right), the only way is to photoexcite the singlet state of an organic chromophore followed by ISC (and RISC for TADF). **b**, Simplified illustration of the spin-exchange coupling-induced direct triplet excitation and emission of an organic chromophore on a LnNC. An unpaired electron of Ln 4*f* orbital will flip accordingly to maintain the total spin of the hybrid upon photoexcitation, leading to the spin-allowed S_0_ → T_1_ transition of the organic chromophore. Spin-exchange coupling promotes nanosecond-timescale phosphorescence, as the radiative decay is now spin-allowed.

The generation and manipulation of triplet states in organic chromophores is crucial for the advancement of optoelectronics^1,2^, photochemical reactions^3,4^ and photo-induced theranostics^5,6^. However, most organic chromophores have a singlet ground state (S_0_), which renders the direct photoexcitation to a triplet state forbidden due to spin-selection rules^7,8^. The radiative recombination of the triplet state via ‘spin-forbidden’ phosphorescence is also very inefficient. Thus, organic triplet states are generally regarded as ‘dark states’. Luminescent harvesting of triplet excitons, by either heavy-metal-induced phosphorescence^9,10^ or thermally activated delayed fluorescence (TADF)^11–13^, relies on intersystem crossing (ISC) or reverse ISC (RISC) (Fig. 1a). Typical ISC processes, however, suffer from energy loss due to the energy gap between the lowest excited singlet state (S_1_) and excited triplet state (T_*n*_). Moreover, the slow ISC and forbidden T_1_ → S_0_ transition endow the organic triplet state with a long lifetime, where nonradiative decay and oxygen quenching become competitive.

To brighten organic triplet states, numerous methods—including crystallization^14–16^ and embedding organics in rigid hosts^17,18^—have been established to realize room-temperature phosphorescence (RTP) in the solid state. Although effective, these methods rely on a crystal lattice to restrict molecular motion and oxygen exposure, which limits their processability and their applications in devices and biological therapeutics. Moreover, most of the luminescence lifetimes of these materials are in the microsecond^19^ to millisecond range, which is not suitable for many applications and also means that nonradiative quenching can be competitive with emission. Consequently, the development of novel approaches to make the organic triplet state bright, especially in solution under ambient conditions, is an important challenge—one that could find a number of use cases.

In this Article we report a simple, straightforward and universal method to brighten the triplet state of organic chromophores by attaching them to the surface of lanthanide-doped nanocrystals (LnNCs) to form hybrid organic–LnNC systems. We show that this allows direct excitation of triplet excitons from the ground state of the system and RTP in solution. The timescale for triplet emission is also reduced to nanosecond timescales, which could allow it to outcompete nonradiative quenching processes.

## Results and discussion

LnNCs have been shown to interact with organic triplet states^20–23^. Energy transfer between the organic chromophore and Ln ions in the NCs has been shown to enhance the emission of Ln ions^24–26^. Recently, we have shown that in LnNCs–organic blend films, the S_0_ → T_1_ transition of organic molecules close to the surface of LnNCs becomes partially allowed. It was proposed that this occurred due to a spin-exchange coupling mechanism^20^. Here we use this mechanism to directly excite the classically ‘dark’ triplet states of organic molecules and obtain phosphorescence in organic–inorganic nanohybrids.

The mechanism for excitation and emission in these systems is shown in Fig. 1b. This mechanism involves the coupling of the spin of unpaired 4*f* electrons of the Ln ion (shown by the red arrow) with the molecular spins. For this coupled spin system, the S_0_ → T_1_ transition becomes allowed, as the spin flip involved in this process is balanced by a spin flip of the unpaired 4*f* electron, leading to no net change of the spin state of the coupled system.

### Spin-exchange coupling-promoted RTP in solution

**Fig. 2:**
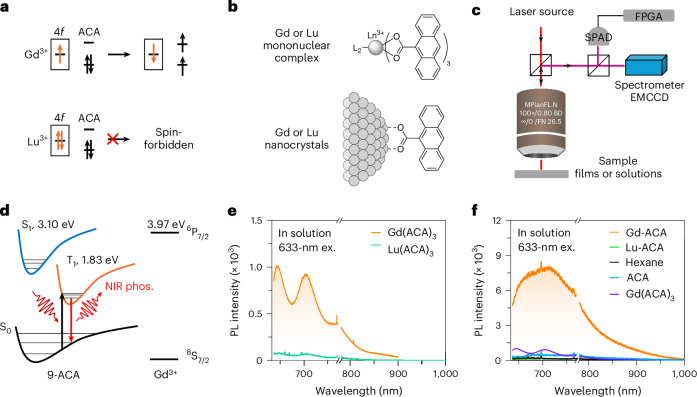
Direct triplet excitation and emission in hybrid organic–inorganic NCs. **a**, Schematic representation of the unpaired 4*f* electron of Gd^3+^ and the fully paired 4*f* electron of Lu^3+^ with frontier orbitals of 9-ACA to emphasize the effect of unpaired 4*f* electrons. **b**, Schematic illustration of the Ln(ACA)_3_ mononuclear complex and Ln–ACA hybrid. **c**, Simple illustration of the microscope set-up. Film and solution samples are placed under the objective lens with well-focused laser, and emission is collected and measured with a single-photon avalanche photodetector (SPAD) for time-correlated single-photon-counting measurements on a field-programmable gate array (FPGA), or on a spectrometer with an electron-multiplying charge-coupled device (EMCCD) camera. For steady-state measurements, a continuous-wave 633-nm laser is used. For lifetime measurements, a pulsed 640-nm laser is used. **d**, Simplified energy levels of 9-ACA and Gd^3+^. Black and red arrows indicate excitation and emission, respectively. **e**, PL spectra of Gd(ACA)_3_ and Lu(ACA)_3_ in DMF under ambient conditions. **f**, PL spectra of Gd–ACA, Lu–ACA (25 mg ml^−1^), ACA (1 mg ml^−1^ in hexane/tetrahydrofuran 9:1, vol/vol) solutions and the solvent hexane under ambient conditions. The normalized Gd(ACA)_3_ PL spectrum is added for comparison of the emission intensity. The excitation wavelength for all spectra is 633 nm. Note that the Raman peaks of hexane and DMF between 770–780 nm are cut for clarity. PL intensity is normalized according to the UV absorbance of coordinated ACA. Source data

Our systems consist of organic chromophores attached to the surface of Gd^3+^-doped NCs in solution. We excited the T_1_ state of the organic chromophore (via the mechanism discussed earlier) using an appropriate long-wavelength laser, which does not excite the higher-energy singlet state of the chromophore. As a control, we used hybrids with organic chromophores attached to the surface of Lu^3+^-doped NCs. In contrast to Gd^3+^, which has seven unpaired electrons in 4*f* orbitals, all electrons are paired in the 4*f* orbitals of Lu^3+^. Therefore, as shown in Fig. 2a, there are no unpaired electrons that can interact with the organic chromophore and thus Lu^3+^ cannot boost the S_0_ → T_1_ transition through spin-exchange coupling.

Anthracene-9-carboxylic acid (ACA) was selected as a model organic chromophore due to its high-lying S_1_ state (~3.10 eV) and relatively low T_1_ state (~1.83 eV)^27^. We envisaged that the large energy gap between its S_1_ and T_1_ states prevents the excitation of S_1_ when a photon energy that is slightly larger than T_1_ is used for direct triplet excitation. Oleic acid (OA)-coated NaGdF_4_ NCs (denoted GdNCs) were prepared through well-established methods with minor modifications^28^. Transmission electron microscopy (TEM) images confirmed their monodispersed feature with an average diameter of 7 nm (Supplementary Fig. 1). ACA was attached onto the surface of GdNCs to form NaGdF_4_@9-ACA hybrids (denoted Gd–ACA) via a passive ligand-exchange method. A detailed description of this method is provided in the Supplementary Information. As solvents we used hexane and *N*,*N*-dimethylformamide (DMF). We note that anhydrous solvents were used, but no further purging was done to remove dissolved oxygen. As a further control, we investigated lanthanide mononuclear complexes (denoted Ln(ACA)_3_; Fig. 2b). The complexes were prepared by mixing precursor solutions of Ln ions and potassium anthracene-9-carboxylate in a 1:3 molar ratio^29^. Figure 2c shows a simple illustration of the microscope set-up used to measure both the photoluminescence (PL) and emission decay profile for all samples in the study.

We began by investigating the lanthanide mononuclear complexes. As shown in Fig. 2e, the complex Gd(ACA)_3_ in DMF shows phosphorescence upon excitation of 633 nm (1.96 eV) in air, whereas the analogue Lu(ACA)_3_ has much weaker emission under the same conditions. The emission spectrum matches well the phosphorescence spectrum of crystalline ACA and ACA in cryogenic frozen solution (Supplementary Figs. 27 and 36). Because the lowest energy level of Gd^3+^ (^6^P_7/2_ → ^8^S_7/2_, 3.97 eV) is too high in energy to be excited (Fig. 2d)^30^, all PL must originate from the ACA triplet, implying that the spin-exchange coupling is effective at inducing phosphorescence following direct triplet excitation.

We next investigated organic–LnNC hybrids. As shown in Fig. 2f, although Gd(ACA)_3_ is phosphorescent, the emission is much weaker than that of the Gd–ACA hybrid (orange line) after normalization by UV absorbance. The phosphorescence emission intensity of the Gd(ACA)_3_ complex at 708 nm is nine times lower than the phosphorescence emission intensity of the Gd–ACA hybrid system. We propose that the ACA triplet on the NC surface has more chances to couple with multiple Gd^3+^ ions, strengthening the spin-exchange coupling in comparison to the mononuclear complex (vide infra). Therefore, incorporation of LnNCs instead of Ln complex is a more efficient way to brighten the triplet of organic chromophores. As further shown in Fig. 2f, negligible emission is observed for pristine ACA dissolved in hexane/tetrahydrofuran (9:1, vol/vol) under ambient conditions (Fig. 2f, blue line). This shows that phosphorescence cannot be realized in solution because of dissolved oxygen and interactions between solvent molecules and the chromophore accelerating nonradiative decay. This is a key hurdle for most organic systems in attempts to yield RTP. In contrast, our Gd–ACA hybrid system in solution exhibits phosphorescence under ambient conditions. The large emission intensity increases between ACA phosphorescence spectra before and after coordination with GdNCs suggest that the S_0_ → T_1_ transition becomes partially allowed and can be directly excited on the surface of GdNCs. Moreover, phosphorescence from such direct triplet excitation is less sensitive to either oxygen or solvent molecules compared with the conventional ISC-phosphorescence mechanism. The phosphorescence quantum yield (*Φ*_P_) of Gd–ACA was calculated to be >2.7% via a relative quantum yield procedure using 6,13-bis(tri-isopropylsilylethynyl)pentacene-2-carboxylic acid (TipsPC) as a reference compound (details of *Φ*_P_ measurements are provided in Supplementary Section 10). This compares to quantum yields of zero for phosphorescence from most organic molecules in solution.

Figure 2f also shows that for NaLuF_4_ NCs with attached ACA (denoted Lu–ACA), no phosphorescence is observed (green line). The atomic number (*Z*) of _71_Lu is larger than that of _64_Gd, so the lack of triplet emission for Lu–ACA excludes the conventional heavy-atom effect that is frequently employed to promote spin–orbital coupling for phosphorescent materials^31,32^. This clearly proves that the our spin-exchange coupling mechanism achieved by unpaired electrons in Ln is a more efficient way to yield triplet emission of organic chromophores than the traditional spin–orbital coupling. No emission is observed when the solvent hexane is excited at 633 nm (Fig. 2f, black line), indicating that the emissions do not originate from any solvent impurities.

More insight into the spin-exchange coupling mechanism was obtained via highly correlated wavefunction-based computational studies (Supplementary Figs. 39 and 40). The calculations indicate a sizeable contribution (by ~20%) of the Gd^3+^ orbitals to the ACA triplet wavefunction, enabling the double spin-flip mechanism. Direct optical absorption into the triplet state is then spin-allowed and borrows intensity from the singlet state, resulting in an ~5 M^−1^ cm^−1^ extinction coefficient, in fair agreement with experiments.

### Studies of energy transfer and shell-thickness dependence

**Fig. 3:**
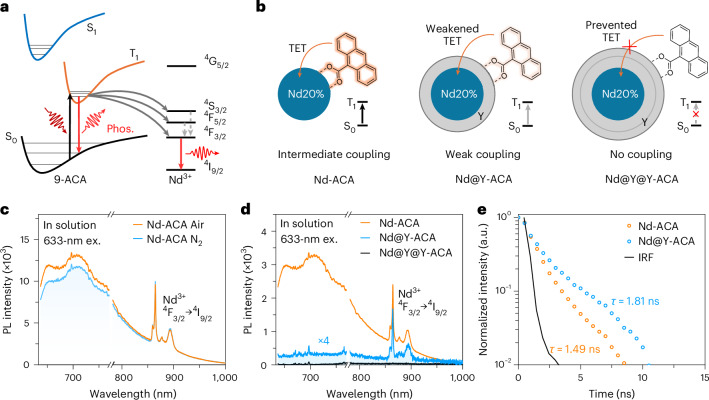
TET and shell-thickness dependence of triplet emission. **a**, Simplified energy levels of ACA and Nd^3+^ in Nd–ACA hybrid NCs. Black, red, pale and dashed arrows indicate excitation, emission, TET and nonradiative conversion, respectively. **b**, Schematic representation of comparisons between Nd–ACA, Nd@Y–ACA and Nd@Y@Y–ACA. Increasing shell thickness weakens coupling between 9-ACA and the core, which makes the S_0_ → T_1_ transition less allowed (indicated by paling arrows) and lower triplet emission (indicated by diminishing glowing) as well as lower Nd emission (indicated by weakened TET). **c**, PL spectra of Nd–ACA (25 mg ml^−1^) solutions in air and under an inert N_2_ atmosphere. The slightly raised emission of Nd–ACA in air is because the air-bubbling process increased the concentration via evaporation. **d**, PL spectra of Nd–ACA, Nd@Y–ACA and Nd@Y@Y–ACA (25 mg ml^−1^) solutions under ambient conditions. The emission intensity of Nd@Y–ACA is multiplied by four for clarity. The excitation wavelength for all spectra is 633 nm. Note that the Raman peak of hexane at 776 nm is cut for clarity. PL intensity is normalized according to the UV absorbance of coordinated ACA. **e**, Emission decay of Nd–ACA (orange dots) and Nd@Y–ACA (blue dots) at 650–750 nm in solution upon pulsed 640-nm excitation. Source data

We investigated the coupling distance for the Ln ions and triplet excitons by careful control of the distance between them. For this, we designed NaGdF_4_:Nd20% NCs with attached ACA (Nd–ACA). We used Nd emission as a marker for energy transfer from the ACA triplet to the LnNCs^33^. Figure 3a presents a schematic of the emission mechanism for Nd^3+^ ions in the Nd–ACA system, where the emission is sensitized by triplet energy transfer (TET) from ACA, following direct excitation of the triplet excitons at 633 nm. The emission of Nd–ACA has a linear power dependence, ruling out the possibility of conventional triplet–triplet annihilation or two-photon absorption to generate a singlet exciton (Supplementary Fig. 33). Figure 3c shows the PL spectra of Nd–ACA in hexane in air or under an inert atmosphere. Both T_1_ emission of ACA and Nd^3+^ (^4^F_3/2_ → ^4^I_9/2_) emission in the near-infrared (NIR) are clearly recorded. A slight emission enhancement of Nd–ACA in air occurs as the air-bubbling procedure removes hexane solvent, slightly increasing the solution’s concentration. Furthermore, the emission ratios between Nd^3+^ at 864 nm and the ACA T_1_ state at 708 nm (*I*_864/708_) are determined to be 0.85 and 0.73 in N_2_ and air, respectively, indicating that there is some quenching of the TET from the ACA triplet to Nd^3+^ in the presence of dissolved oxygen in the system, but that this emission is not completely quenched.

We next fabricated a series of core–shell structures (NaGdF_4_:Nd20%@NaYF_4_) containing a NaGdF_4_:Nd20% core and different yttrium shell thicknesses achieved by continuous epitaxial growth of another yttrium shell onto the existing core–shell (Fig. 3b)^34^. ACA was attached onto the surface of the core–shell and core–shell–shell NCs (denoted Nd@Y–ACA and Nd@Y@Y–ACA, respectively) via passive ligand exchange. The yttrium shell was chosen because Y^3+^ cannot promote the S_0_ to T_1_ transition like Gd^3+^ (or Nd^3+^) due to its empty 4*f* orbitals; that is, there can be no spin-exchange coupling between ACA and Y. Therefore, we hypothesize that increasing the distance between the chromophore and core (which contains Gd^3+^ and Nd^3+^ and hence unpaired electrons that support the spin-exchange mechanism) will reduce the efficiency of direct excitation of T_1_ of coordinated ACA, resulting in gradually diminishing emission.

Figure 3d shows the thickness-dependent PL of these hybrids in hexane solution under ambient conditions upon excitation at 633 nm. Compared with the emission of Nd–ACA (orange line), the overall emission intensity of Nd@Y–ACA (blue line) decreases dramatically. The relative intensity *I*_864/708_ of Nd@Y–ACA was calculated to be 5.11, much higher than that of Nd–ACA (0.73). The much lower ACA triplet emission is consistent with a weaker spin-exchange coupling in the presence of the thin yttrium shell (~3.5 nm). In contrast, emission from the Nd^3+^ is still observed and is not greatly diminished compared to the Nd–ACA (orange line). This suggests that the TET processed from the coordinated ACA to Nd^3+^ is less efficient, but not completely shut down by the presence of the thin yttrium shell. Additionally, the 633-nm excitation is close to the Nd^3+^ absorption, which could increase the emission of Nd^3+^ in the NIR range. This is consistent with recent results, which show that triplets can be transferred from organic molecules into LnNCs even in the presence of a short aliphatic spacer^28^.

We further increased the yttrium shell thickness as shown in Fig. 3b (right panel). This had the effect of quenching the emission from both ACA T_1_ and Nd^3+^ (Fig. 3d, black line). This shows that the thick yttrium shell completely prevents spin-exchange coupling between Ln and the triplet of ACA.

Emission lifetime decay profiles for the T_1_ of ACA on the surface of NdNCs and Nd@Y NCs were recorded by time-correlated single-photon counting (TCSPC) in the presence of appropriate filters. Rather than the micro- to millisecond-level lifetime of conventional organic phosphorescence, the decay profiles for 650–750 nm reveal lifetimes of 1.49 ns and 1.81 ns for Nd–ACA and Nd@Y–ACA, respectively (Fig. 3e). The emission of Nd^3+^ in the NIR range has a microsecond-timescale lifetime (Supplementary Fig. 30). The nanosecond-lifetime phosphorescence in the Nd–ACA hybrid suggests that the S_0_ → T_1_ transition is partially allowed, which leads to a triplet population via direct excitation from S_0_, instead of slow ISC from S_1_, and fast emission decay.

With the core–shell Nd@Y–ACA system, the average lifetime of T_1_ emission of ACA is longer than that of the ACA T_1_ on core-only NCs. This suggests that the S_0_ → T_1_ transition is less allowed due to weakened spin-exchange coupling in the presence of the yttrium shell. The lifetime of T_1_ emission of Gd–ACA was measured to be 2.03 ns (Supplementary Fig. 31). As no energy transfer happens between Gd^3+^ (without energy levels to receive triplet energy) and ACA, the shortened lifetime solely results from spin-exchange coupling. Compared with the lifetime of Gd–ACA, Nd–ACA provides a shortened lifetime of 1.49 ns due to spin-exchange coupling, as well as TET between ACA and Nd^3+^. The lifetime changes match well with the triplet rise and decay in transient absorption spectra, where the triplet rises and decays slowly on Lu–ACA, but more quickly on Gd–ACA due to spin-exchange coupling. On Nd–ACA, decay of the ACA triplet is further accelerated by TET (Supplementary Fig. 32).

Given the fact that the triplet lifetime is in the nanosecond range, the triplet emission should not be sensitive to the surrounding environments, in line with oxygen-insensitive phosphorescence in solution. We infer that such robust phosphorescence holds great potential in biological applications, as most NIR chromophores suffer from energy transfer to oxygen, causing ^1^O_2_-induced photobleaching and unwanted biological side effects under long-term irradiation^35–37^. In our hybrid system, reduced oxidation-induced photobleaching enables persistent NIR emission in solution, with better biocompatibility.

### Chromophore and lanthanide universality

**Fig. 4:**
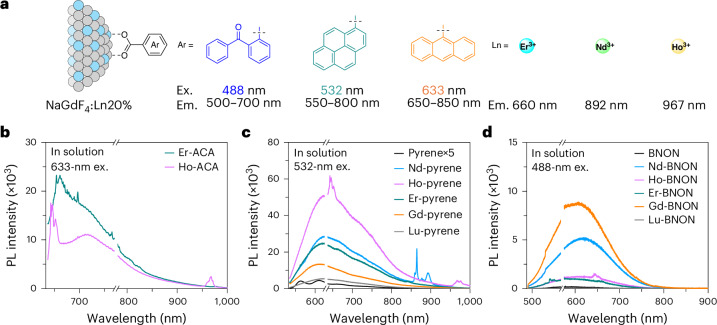
Triplet emissions from a range of coordinated chromophores. **a**, Organic chromophores and doped Ln^3+^ ions used for testing the substrate scope. **b**, PL spectra of Ln–ACA solutions. The excitation wavelength is 633 nm. **c**, PL spectra of Ln–pyrene system solutions. The excitation wavelength is 532 nm. **d**, PL spectra of Ln–BNON system solutions. The excitation wavelength is 488 nm. All spectra were measured under ambient conditions and calibrated by the UV absorbance of organic chromophores. The concentration for each hybrid was 25 mg ml^−1^ in hexane. The concentration for pristine organic chromophores was 1 mg ml^−1^ in hexane/tetrahydrofuran (9:1, vol/vol). Source data

We next explored the generality of this approach for room-temperature solution-phase phosphoresce via spin-exchange coupling between organic chromophores and LnNCs, by testing different organic molecules to observe triplet emission. A detailed energy diagram of lanthanide doping and organic chromophores is shown in Supplementary Fig. 37. We selected pyrene-1-carboxylic acid (pyrene) and benzophenone-2-carboxylic acid (BNON), whose triplet states can be excited by green (532 nm) and blue (488 nm) lasers, respectively (Fig. 4a). The palette of Ln elements was also extended by doping 20% of Er or Ho in NaGdF_4_. These Ln–organic hybrids (denoted Ln–pyrene and Ln–BNON, respectively) were prepared by the same passive ligand-exchange method. Figure 4b shows PL spectra of Er–ACA and Ho–ACA excited by a 633-nm laser in hexane under ambient conditions. Emission at ~710 nm, as well as nanosecond-timescale decays (Supplementary Fig. 29) from the T_1_ of coordinated ACA, are observed for both hybrids, confirming that doped Ln^3+^ does not affect direct triplet excitation and emission. Additionally, we also recorded emissions with microsecond-timescale decays (Supplementary Fig. 30) from Ho^3+^ at 650 nm (^5^F_5_ → ^5^I_8_) and 970 nm (^5^I_5_ → ^5^I_8_) and Er^3+^ at 660 nm (^4^F_9/2_ → ^4^I_15/2_), which result from TET from ACA.

As shown in Fig. 4c, when a solution of pyrene in hexane/tetrahydrofuran (9:1, vol/vol) is excited by a 532-nm laser under ambient conditions (Fig. 4c), weak emission is observed, in line with the previously reported T_1_ emission for pyrene at ~2.00 eV (620 nm)^27^. After coordination onto LuNCs, the emission at 610 nm is enhanced by 5.88 times (grey line), and by 14.8 times on GdNCs (orange line). We ascribe the emission enhancement of LuNCs to the heavy-atom effect. Overall, the emission enhancement observed must be a combination of spin–orbit coupling (heavy-atom effect, $\propto {Z}^{4}$) enhancement and spin-exchange coupling enhancement. Both are in play, and the spin-exchange enhancement is dominant in all cases considered, as Gd^3+^ nanocrystals with seven unpaired electrons have stronger phosphorescence enhancement than Lu^3+^. A detailed discussion is provided in Supplementary Section 9. Additionally, the emission from pyrene is more complex, and may involve the formation of transient excimers with free-floating molecules in solution as the surface coordination is reversible. Other Ln dopants maintain the strength of spin-exchange coupling, affording bright T_1_ emission. The highest emission intensity is obtained by coordinating pyrene onto HoNCs.

We note that the 532 nm excitation wavelength in Ho-based systems is absorbed not only by the S_0_ → T_1_ transition of coordinating pyrene, but also by the ^5^I_8_ → ^5^F_4_ transition (centred at 535 nm) of Ho^3+^, which is followed by reverse TET from ^5^F_4_ to the T_1_ of pyrene, yielding a higher T_1_ population and highest PL. For Ln emission in the pyrene system, Ho^3+^ emissions at 650 nm (^5^F_5_ → ^5^I_8_) and 970 nm (^5^I_5_ → ^5^I_8_) and Nd^3+^ emission at 864 nm (^4^F_3/2_ → ^4^I_9/2_) are found in the Ho–pyrene and Nd–pyrene solutions, respectively. Ho^3+^ emissions may originate from direct absorption of the 532-nm laser and mutual energy transfer between the T_1_ of surface pyrene and the ^5^F_4_ state, and the Nd^3+^ emission may result from TET to the ^2^G_7/2_ + ^4^G_5/2_ states at 575 nm. Er^3+^ emission at 660 nm (^4^F_9/2_ → ^4^I_15/2_) disappears in the Er–pyrene solution, which could be ascribed to a very fast radiative process of the T_1_ of pyrene as well as a relatively large energy gap between pyrene and Er^3+^.

Figure 4d shows the PL spectra of Ln–BNON solutions under 488-nm excitation. Compared to the negligible emission from pristine BNON and the Lu–BNON hybrid, BNON coordinated onto Gd, Nd, Ho and Er NCs exhibits emission at ~620 nm, with the maximum PL intensity obtained from Gd–BNON. For Ln emissions, our 488 nm excitation elevates Ho^3+^ to its ^5^F_3_ excitation state and Er^3+^ to its ^4^F_5/2_ state, so emissions of Ho^3+^ at 650 nm (^5^F_5_ → ^5^I_8_) and Er^3+^ at 550 nm (^4^S_3/2_ → ^4^I_15/2_) and 660 nm (^4^F_9/2_ → ^4^I_15/2_) are all observed. The emission of Nd^3+^, however, disappears compared with other Nd–organic hybrids, due to inefficient TET. Additionally, the emission does not have an excitation wavelength dependence, as reflected by the very similar emission maxima of Nd–BNON excited at either 488 nm or 532 nm. These results show that the triplet states of coordinated chromophores on the surfaces of Ln NCs with the NaGdF_4_ matrix could be directly excited from their ground states, regardless of triplet-state energies. These triplet states decay radiatively, producing phosphorescence in solution under ambient conditions. Doping different Ln^3+^ ions to NaGdF_4_ maintains the strength of triplet excitation and emission and achieves different TET pathways and Ln emissions.

## Conclusion

In summary, we have demonstrated that, via the spin-exchange coupling mechanism between organic molecules and LnNCs, the excitation and emission of S_0_ → T_1_ on surface-bound molecular chromophores can be achieved directly, even in solution and ambient oxygenated conditions. Conventionally, the triplet state of an organic chromophore is only accessible via an ISC-mediated spin flip, and radiative return to the ground state is forbidden. This makes phosphorescence competitive with nonradiative decay and oxygen quenching, leading to weak phosphorescence in the solid state and no phosphorescence in solution at room temperature. Our strategy utilizes a spin-exchange mechanism, leading to ‘spin-allowed’ triplet-state excitation from the ground singlet state, and fast phosphorescence emission. This allows for much stronger and oxygen-insensitive nanosecond-timescale phosphorescence. The total spin quantum number remains unchanged due to the reversible spin flip of an electron in the 4*f* orbitals of the Ln along with S_0_ → T_1_ excitation and radiative decay. PL measurements confirm that unpaired electrons in Ln 4*f* orbitals are crucial for this transition. No emission is recorded when Lu^3+^ is employed in the matrix on account of its full 4*f* orbitals coupled with ACA, despite its larger atomic number, excluding the conventional heavy-metal effect. The emission from direct triplet excitation also shows a clear distance dependence, as an inert Y^3+^ shell blocks spin-exchange coupling. By investigating different organic chromophores, we also demonstrate that this method works for visible and NIR dyes.

Improved practicality and utility in biological systems can be foreseen with such a triplet excitation and emission strategy. In contrast to most conventional RTP materials with long triplet lifetimes, which require crystallization to minimize nonradiative decay and avoid oxygen quenching, our RTP system works in solution under ambient conditions. Avoiding using fragile crystalline materials as well as environmental tolerance offer processability and great practical potential. Moreover, a redshifted excitation wavelength and NIR phosphorescence render these Ln–organic hybrids suitable for future biological imaging without any risk of singlet oxygen generation and dye bleaching. The triplet of the hybrid is also bright on films, enabling the development of optoelectronic materials and devices.

## Methods

### Preparation of Gd–ACA hybrid via passive ligand exchange

To a solution of ACA in hexane/tetrahyrofuran = 9:1 (1 ml, 1 mg ml^−1^), 1 ml of NaGdF_4_ stock solution (25 mg ml^−1^) was added. The mixture was ultrasonicated for 15 min at room temperature, then kept in the dark overnight. The modified Gd–ACA nanocrystals were precipitated by adding 5 ml of ethanol, then collected by centrifugation. After washing with ethanol twice, the solids were dissolved in 1 ml of hexane for further characterization.

Other organic–inorganic hybrids were prepared with the same method but using Ln-doped nanocrystals, core–shell nanocrystals as the starting material or other organic chromophores as the ligand.

### PL spectro-microscopy

Optical spectra were recorded with a custom-built visible microscope with a motorized stage and spectrometers, and with a commercial Raman microscope. The home-built microscope used a 633-nm laser (Matchbox) to excite lanthanide nanomaterials on quartz or glass coverslips, and collected the emission in reflection mode with a 0.9 NA objective lens with an average power of <100 µW µm^−2^ on the sample. The back-scattered emitted light was filtered through two notch filters (centred at 633 nm, Semrock) before being dispersed on a Shamrock i303 spectrograph with a Newton EMCCD (Andor).

## Online content

Any methods, additional references, Nature Portfolio reporting summaries, source data, extended data, supplementary information, acknowledgements, peer review information; details of author contributions and competing interests; and statements of data and code availability are available at 10.1038/s41557-026-02159-w.

## Supplementary information

Supplementary Information Supplementary Information, including Methods, Materials, Figures and Discussion.

Supplementary Data 1 Computational data (atomic coordinates).

## Source data

Source Data Fig. 2 Unprocessed photoluminescence data.

Source Data Fig. 3 Unprocessed photoluminescence and decay profile.

Source Data Fig. 4 Unprocessed photoluminescence data.

## Data Availability

The data underlying all figures in the main text are publicly available from the University of Cambridge repository at 10.17863/CAM.128908 (ref. ^38^). Source data are provided with this paper.
