# Supplementary material for: Lanthanide-doped nanocrystals enable organic room-temperature phosphorescence in solution through direct triplet excitation
Source: Nat Chem. 2026 May 25;18(7):1242–8. doi: 10.1038/s41557-026-02159-w (PMC13323090; doi:10.1038/s41557-026-02159-w)
Supplement: Supplementary file 1 — Supplementary Information, including Methods, Materials, Figures and Discussion. [file 41557_2026_2159_MOESM1_ESM.pdf]

nature chemistry

Supplementary information

https://doi.org/10.1038/s41557-026-02159-w

# Lanthanide-doped nanocrystals enable organic room-temperature phosphorescence in solution through direct triplet excitation

In the format provided by the authors and unedited

# Table of contents


**1. Materials and methods** ............................................................ 2

**Synthesis of Gd(ACA)$_3$ mononuclear complex** ............................................................ 2

**Synthesis of $NaGdF_4$ nanocrystals** ............................................................ 2

**Synthesis of core-shell $NaGdF_4$:Nd20%@$NaYF_4$ (Nd@Y) nanocrystals** ............................................................ 3

**Synthesis of core-shell-shell $NaGdF_4$:Nd20%@$NaYF_4$@$NaYF_4$ (Nd@Y@Y) nanocrystals** ..... 3

**Preparation of Gd-ACA via passive ligand exchange** ............................................................ 3

**Transmission electron microscopy (TEM)** ............................................................ 4

**UV-vis spectroscopy** ............................................................ 4

**Photoluminescence spectro-microscopy.** ............................................................ 4

**Time-resolved single-photon counting microscopy.** ............................................................ 5

**2. TEM images of nanocrystals** ............................................................ 6

**3. UV-vis spectra** ............................................................ 10

**4. Phosphorescence of ACA crystals and the Nd-ACA film** ............................................................ 17

**5. TCSPC data** ............................................................ 18

**6. Transient absorption data** ............................................................ 21

**7. Power-dependent emissions** ............................................................ 23

**8. Surface loading calculation** ............................................................ 24

**9. Effects of spin-orbital and spin-exchange couplings** ............................................................ 26

**10. Phosphorescence quantum yield ($\Phi_P$) and molar absorption coefficient ($\varepsilon$) of Gd-ACA in solution** ............................................................ 28

**Lower-bound estimation of $\Phi_P$** ............................................................ 28

**Upper-bound estimation of $\varepsilon$** ............................................................ 30

**11. Cryogenic frozen solution phosphorescence measurements** ............................................................ 31

**12. Energy diagram** ............................................................ 33

**13. Wavelength-dependent emission spectra** ............................................................ 34

**14. Computational studies of spin-exchange coupling** ............................................................ 35

**15. References** ............................................................ 41

## 1. Materials and methods

Sodium hydroxide (>98%), ammonium fluoride (99%), gadolinium(III) acetate ($Gd(OAc)_3 \cdot xH_2O$, >99.9%), neodymium(III) acetate ($Nd(OAc)_3 \cdot xH_2O$, >99.9%), holmium acetate ($Ho(OAc)_3 \cdot xH_2O$, >99.9%), erbium acetate ($Er(OAc)_3 \cdot xH_2O$, >99.9%), yttrium acetate ($Y(OAc)_3 \cdot xH_2O$, >99.9%), sodium trifluoroacetate (NaTFA, 99%), trifluoroacetic acid (TFA, 95%) and all solvents used including oleic acid (OA, 90%) and 1-octadecene (ODE, 90%) were purchased from Sigma Aldrich. All inorganic salts and solvents were used as received unless stated otherwise. Anthracene-9-carboxylic acid (9-ACA, 99%), pyrene-1-carboxylic acid (Pyrene, 99%) and benzophenone-2-carboxylic acid (BNON, 99%) were purchased from Sigma Aldrich and recrystallized twice using ethanol before any material preparation and photophysical measurement to eliminate any impurity. The UV epoxy glue is purchased from Blufixx, with the product name BLUFIXX XPRESS UV SUPERGLUE.

### Synthesis of $Gd(ACA)_3$ mononuclear complex

The mononuclear complex was synthesized using a modified procedure[1]. Briefly, 0.3 mmol of KOH and 0.3 mmol of 9-ACA were mixed in 10 mL of water, producing a clear light-yellow solution of potassium anthracene-9-carboxylate. Under vigorous stirring, aqueous solutions of 0.1 mmol of $Gd(NO_3)_3 \cdot xH_2O$ and 0.3 mmol of potassium anthracene-9-carboxylate were mixing. White precipitate formed immediately, which was collected by centrifugation. The solids were washed with ethanol three times, collected by centrifugation and dried in vacuum as the product. The final product was dissolved in 5 mL of DMF for further characterizations.

The $Lu(ACA)_3$ mononuclear complex was prepared in the same method but using $LuCl_3 \cdot xH_2O$ as the starting material.

### Synthesis of $NaGdF_4$ nanocrystals

1.0 mmol of $Gd(OAc)_3 \cdot xH_2O$ was suspended in 6 mL of OA and 10 mL of ODE. The mixture was heated to 140 °C for 1 h under $N_2$ atmosphere, yielding a clear $Gd^{3+}$ precursor solution. To this precursor solution, 8 mL of a methanol solution containing 0.96 mmol of $NH_4F$ and 0.6 mmol NaOH wad added. The pale suspension was heated at 70 °C for 45 min under $N_2$ flow to eliminate methanol. Subsequently, the mixture was heated to 300 °C for 1 h under $N_2$

atmosphere. After cooling to room temperature, 20 mL of ethanol was added to precipitate nanocrystals. The precipitate after centrifugation was washed with ethanol twice, affording white solids as the product that were dissolved in hexane at 25 mg/mL for further characterizations[2].

$NaLuF_4$ nanocrystals were prepared in the same way but using $Lu(OAc)_3 \cdot xH_2O$ as the starting material. $NaGdF_4$:Ln20% (Ln = Nd, Ho, Er) nanocrystals were prepared with the same procedure but using a mixture of 0.8 mmol of $Gd(OAc)_3 \cdot xH_2O$ and 0.2 mmol of $Ln(OAc)_3 \cdot xH_2O$ as the starting material.

### Synthesis of core-shell $NaGdF_4$:Nd20%@$NaYF_4$ (Nd@Y) nanocrystals

The shelling process was modified from a published procedure[3]. The shell precursors containing 1.0 mmol of $Y(TFA)_3$ and 1.8 mmol of NaTFA were mixed in 4 mL of OA and 6 mL of ODE. The salts were fully dissolved upon heating to 120 °C for 30 min under $N_2$ atmosphere. After cooling to room temperature, the solution containing shell precursors was loaded into a syringe pump for subsequent injection. 0.4 mmol of the core nanocrystal $NaGdF_4$:Nd20% was dissolved in 8 mL of OA and 8 mL of ODE. The core solution was heated to 120 °C for 30 min under $N_2$ flow to eliminate any water residue. After that, the core solution was further heated to 280 °C and the shell precursors were injected at a rate of 6.5 mL/h. After injection, the solution was heated to 325 °C for 20 min. After cooling to room temperature, 20 mL of ethanol was added to precipitate nanocrystals. The precipitate after centrifugation was washed with ethanol twice, affording white solids as the $NaGdF_4$:Nd20%@$NaYF_4$ (Nd@Y) that were dissolved in hexane for further characterizations.

### Synthesis of core-shell-shell $NaGdF_4$:Nd20%@$NaYF_4$@$NaYF_4$ (Nd@Y@Y) nanocrystals

The core-shell-shell Nd@Y@Y was prepared using the similar procedure. In this step, Nd@Y nanocrystals were applied as the core rather than $NaGdF_4$:Nd20%.

### Preparation of Gd-ACA via passive ligand exchange

To a solution of ACA in hexane/THF = 9:1 (1 mL, 1 mg/mL), 1 mL of the $NaGdF_4$ stock solution (25 mg/mL) was added. The mixture was ultrasonicated for 15 min at room

temperature and then kept in dark overnight. The modified Gd-ACA nanocrystals were precipitated by adding 5 mL of ethanol and collected by centrifugation. After washed with ethanol twice, the solids were dissolved in 1 mL of hexane for further characterizations.

Other organic-inorganic hybrids were prepared with the same method but using Ln-doped nanocrystals, core-shell nanocrystals as the starting material or other organic chromophores as the ligand.

### Transmission electron microscopy (TEM)

TEM images were recorded on an FEI Tecnai F20 instrument at 200 kV accelerating voltage. The stock solutions of nanocrystals were diluted with hexane and then dropcasted onto 200-mesh Cu grids (Agar AGS160).

### UV-vis spectroscopy

All UV-vis spectra were recorded on a KHMD Shimadzu UV-3600i Plus instrument. The spectra were recorded under ambient conditions without inert gas purge. Ln-organic hybrids were measured with their stock solutions without dilution. The absorbance of pure organic chromophores was adjusted to a proper range with hexane dilution. Intensity of photoluminescence of each sample was normalized based on absorbance of organic chromophores in order to compare the emission intensity.

### Photoluminescence spectro-microscopy

Optical spectra were recorded with custom-built visible microscope with a motorized stage and spectrometers, and with a commercial Raman microscope. The home-built microscope used a 633 nm laser (Matchbox) to excite lanthanide nanomaterials on quartz or glass coverslips, and collected the emission in reflection mode with a 0.9 NA objective lens with <100 $\mu W\ \mu m^{-2}$ average power on the sample. The back-scattered emitted light was filtered through two notch filters (centred at 633 nm, Semrock) before being dispersed on a Shamrock i303 spectrograph with a Newton electron multiplying charge coupled device (Andor).

The commercial Raman system (confocal Renishaw InVia microscope with 450 nm, 532 nm, 633 nm, and 785 nm excitation lasers) was used to collect triplet emission spectra in solution

and solid state. The laser power was kept below 1 mW, and the light was collected with a 20X (0.4 NA) objective lens, before being dispersed onto a 1200 lines/mm grating on a CCD.

### Time-resolved single-photon counting microscopy.

Time-resolved photoluminescence was recorded on the home-built microscope with a 640 nm pulsed excitation source (PicoQuant LDH-P-C-400B, driven at 4kHz, 40 kHz and 40MHz, filtered by an angle-tuned 650 nm bandpass). The luminescence was collected with a darkfield objective lens (Olympus, 100X, 0.9 NA) in reflection and filtered with a 650 nm long-pass before routing to a single-photon avalanche photodiode (Micro Photon Devices PDM PD-100-CTD). Additional spectral filters were used to isolate the lifetimes from the lanthanide ion emission and the molecular triplet emission. The arrival times of all photons were continuously recorded with a time-tagged system on a field-programmable gate array board[4], and analysed to obtain the photoluminescence lifetime trace.

## 2. TEM images of nanocrystals

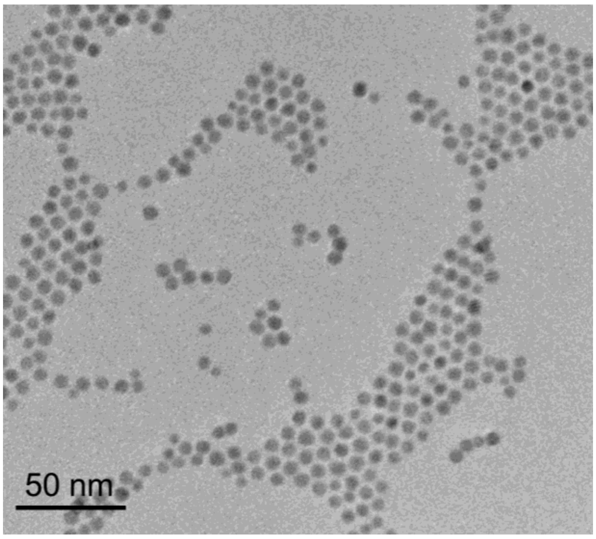


Supplementary Fig. 1. TEM image of $NaGdF_4$ nanocrystals. The scale bar is 50 nm.

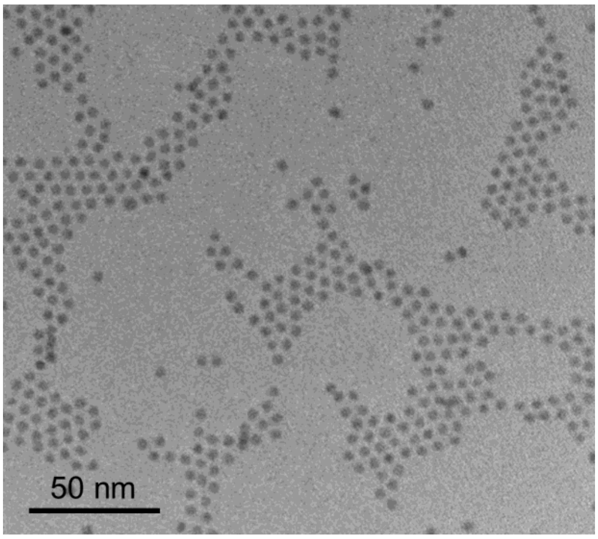


Supplementary Fig. 2. TEM image of $NaLuF_4$ nanocrystals. The scale bar is 50 nm.

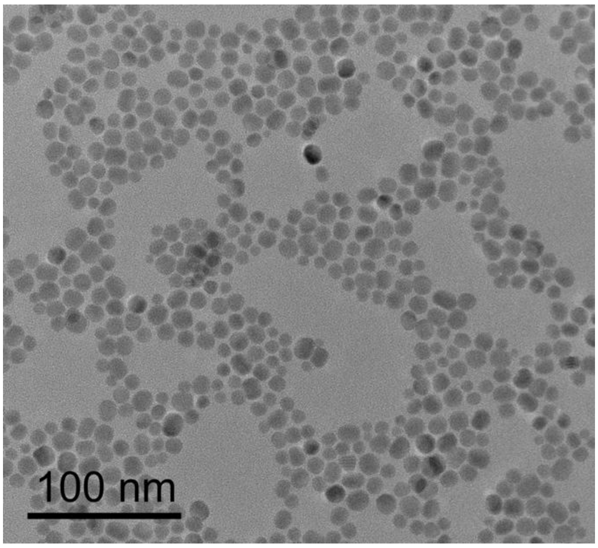


Supplementary Fig. 3. TEM image of $NaGdF_4$:Nd20% nanocrystals. The scale bar is 100 nm.

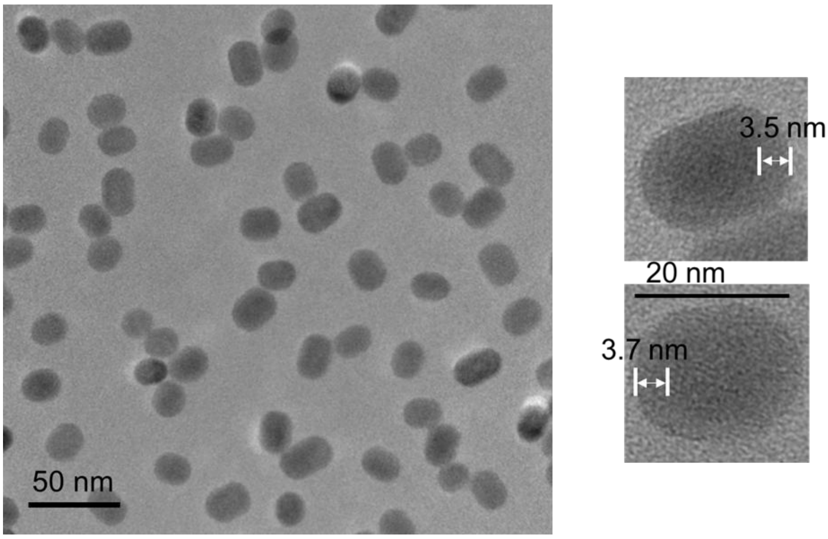


Supplementary Fig. 4. TEM images of $NaGdF_4$:Nd20%@$NaYF_4$ nanocrystals (left) and enlarged nanocrystals to measure shell thickness (right). The average shell thickness is around 3.5 m.

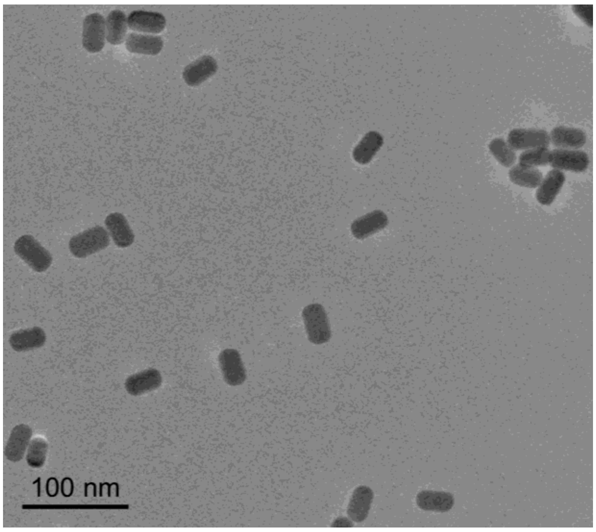


Supplementary Fig. 5. TEM image of $NaGdF_4$:Nd20%@$NaYF_4$@$NaYF_4$ nanocrystals. The scale bar is 100 nm.

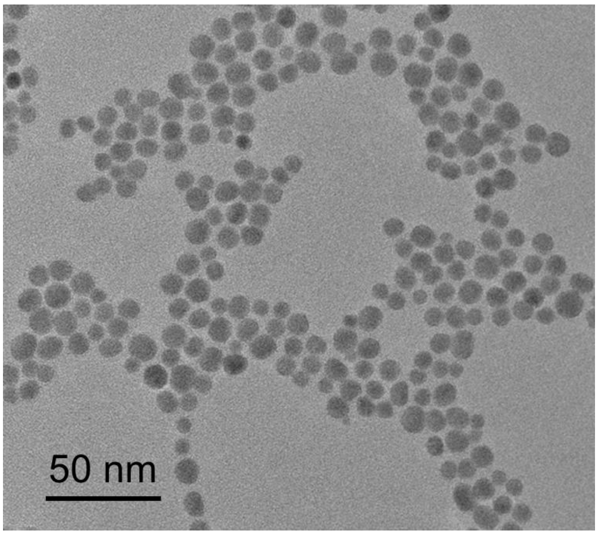


Supplementary Fig. 6. TEM image of $NaGdF_4$:Ho20% nanocrystals. The scale bar is 50 nm.

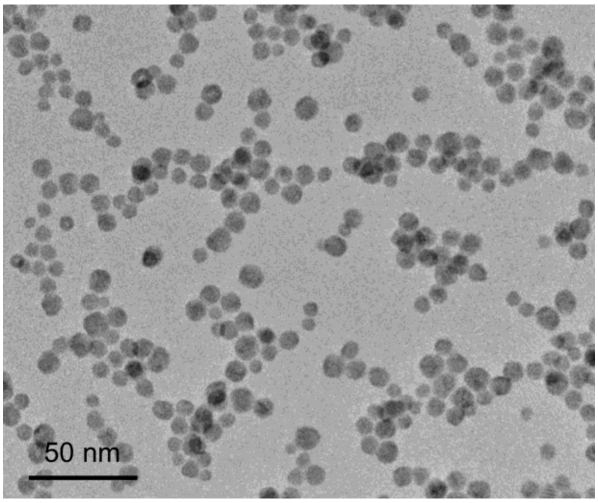


Supplementary Fig. 7. TEM image of $NaGdF_4$:Er20% nanocrystals. The scale bar is 50 nm.

## 3. UV-vis spectra

The UV-vis absorption spectra of organic molecules on the lanthanide nanocrystal surface or in organic-lanthanide complexes were recorded to normalized emission intensity.

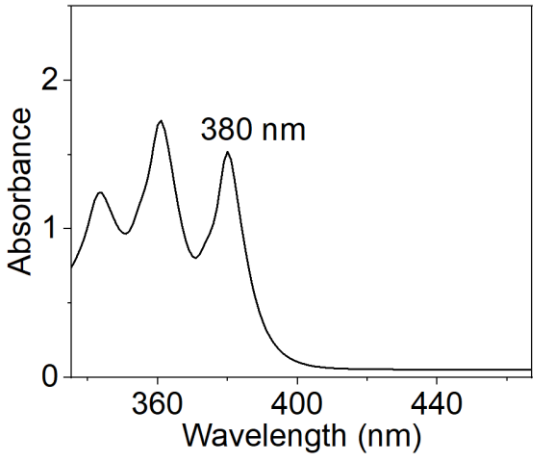


Supplementary Fig. 8. UV-vis spectrum of ACA (1 mg/mL) in THF : hexane = 1:9 (*v*/*v*).

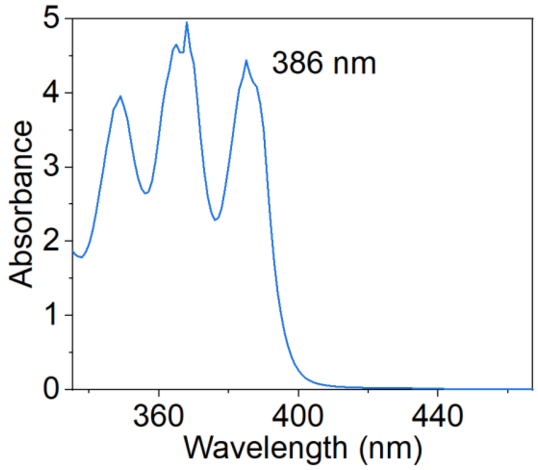


Supplementary Fig. 9. UV-vis spectrum of the as-prepared $Gd(ACA)_3$ complex in hexane.

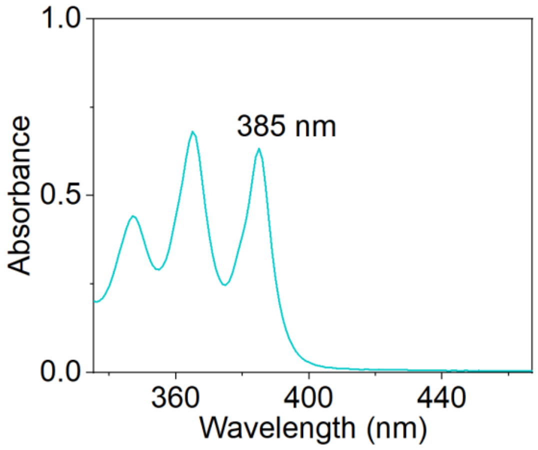


Supplementary Fig. 10. UV-vis spectrum of the as-prepared Lu(ACA)$_3$ complex in hexane.

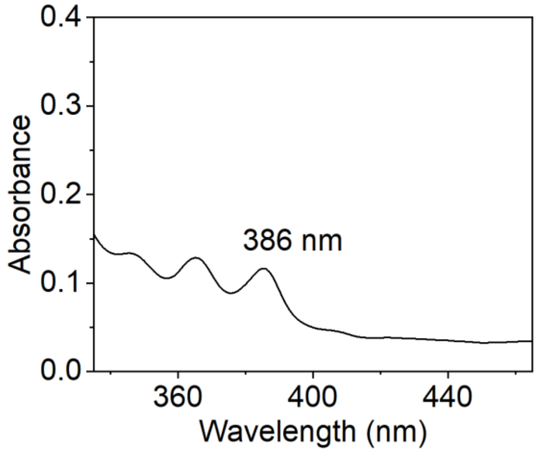


Supplementary Fig. 11. UV-vis spectrum of the as-prepared Gd-ACA hybrid in hexane.

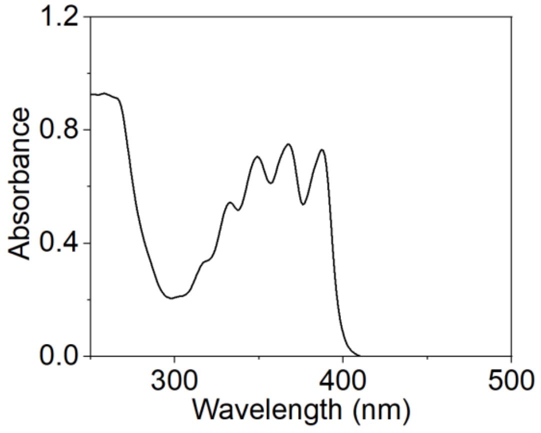


Supplementary Fig. 12. UV-vis spectrum of the as-prepared Nd-ACA hybrid in hexane.

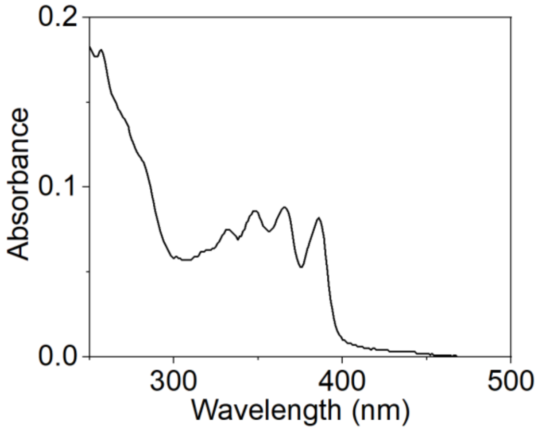


Supplementary Fig. 13. UV-vis spectrum of the as-prepared Er-ACA hybrid in hexane.

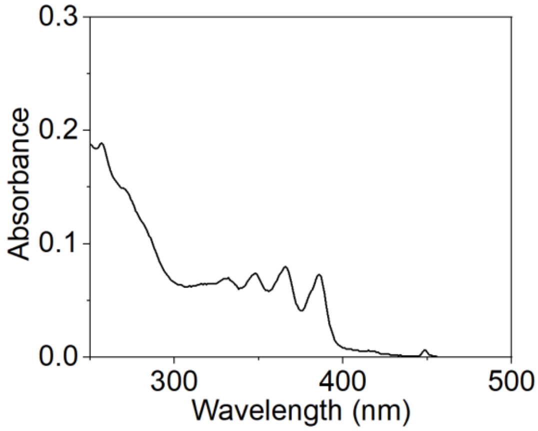


Supplementary Fig. 14. UV-vis spectrum of the as-prepared Ho-ACA hybrid in hexane.

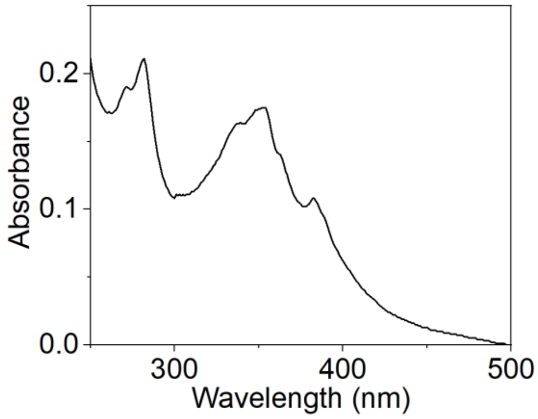


Supplementary Fig. 15. UV-vis spectrum of Pyrene in THF : hexane = 1:9 (*v*/*v*).

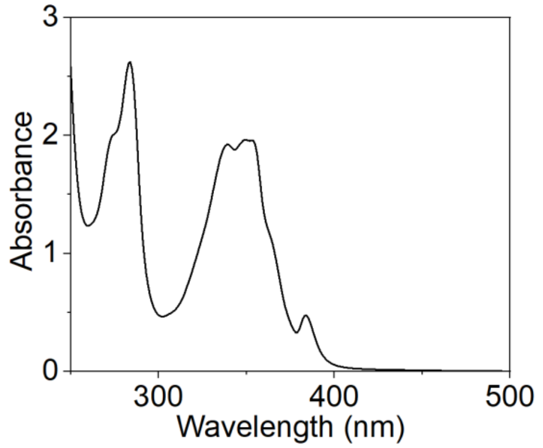


Supplementary Fig. 16. UV-vis spectrum of the as-prepared Gd-Pyrene hybrid in hexane.

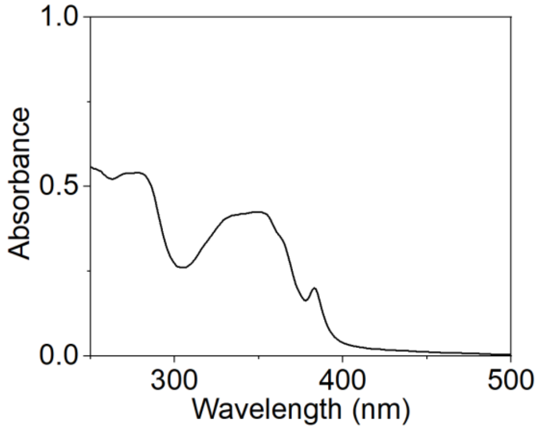


Supplementary Fig. 17. UV-vis spectrum of the as-prepared Lu-Pyrene hybrid in hexane.

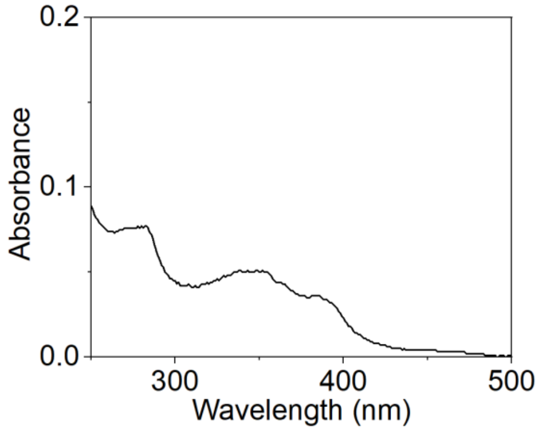


Supplementary Fig. 18. UV-vis spectrum of the as-prepared Nd-Pyrene hybrid in hexane.

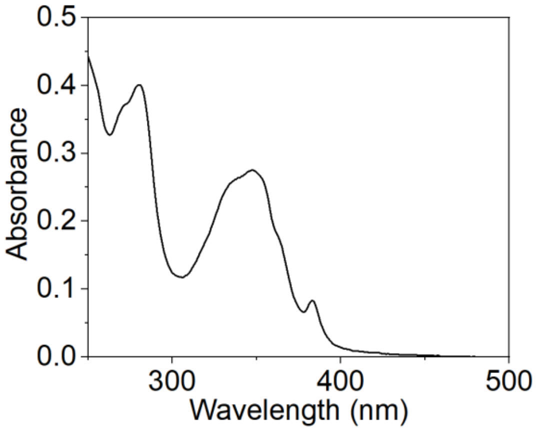


Supplementary Fig. 19. UV-vis spectrum of the as-prepared Er-Pyrene hybrid in hexane.

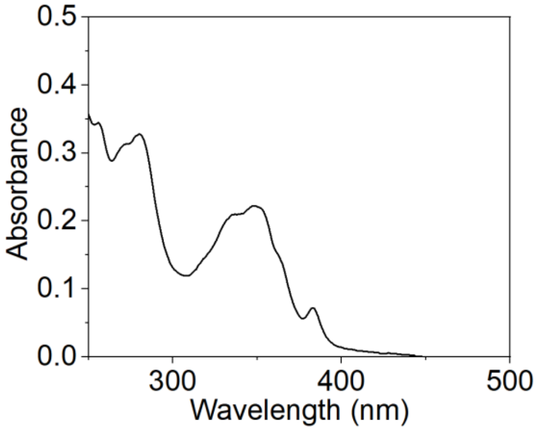


Supplementary Fig. 20. UV-vis spectrum of the as-prepared Ho-Pyrene hybrid in hexane.

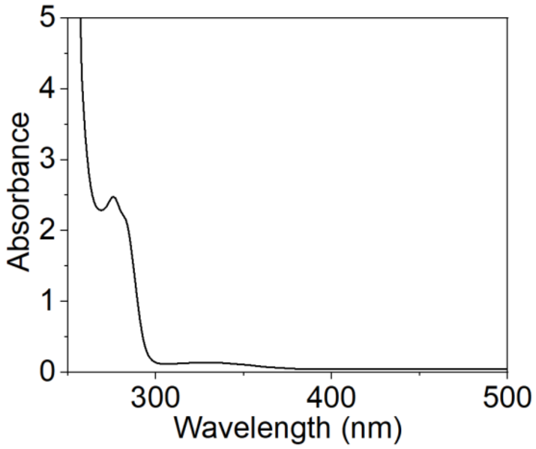


Supplementary Fig. 21. UV-vis spectrum of BNON in THF : hexane = 1:9 (*v*/*v*).

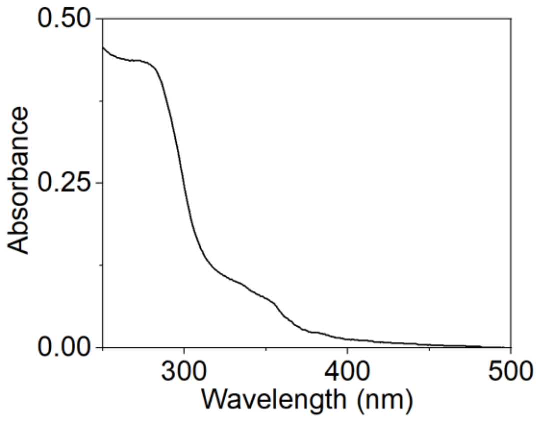


Supplementary Fig. 22. UV-vis spectrum of the as-prepared Gd-BNON hybrid in hexane.

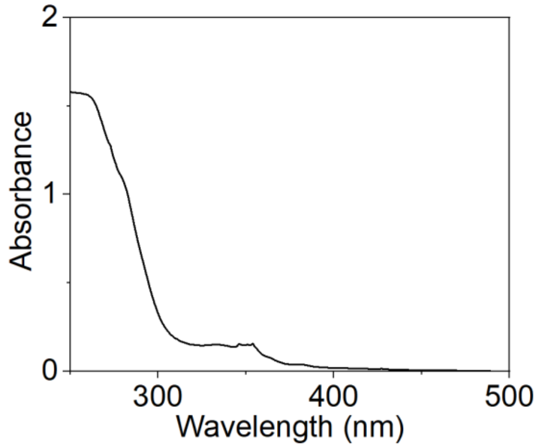


Supplementary Fig. 23. UV-vis spectrum of the as-prepared Lu-BNON hybrid in hexane.

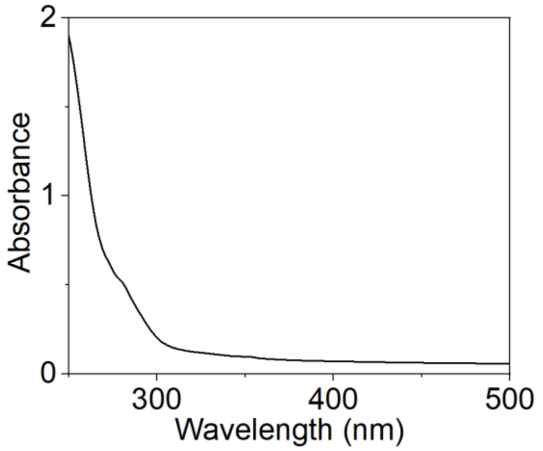


Supplementary Fig. 24. UV-vis spectrum of the as-prepared Nd-BNON hybrid in hexane.

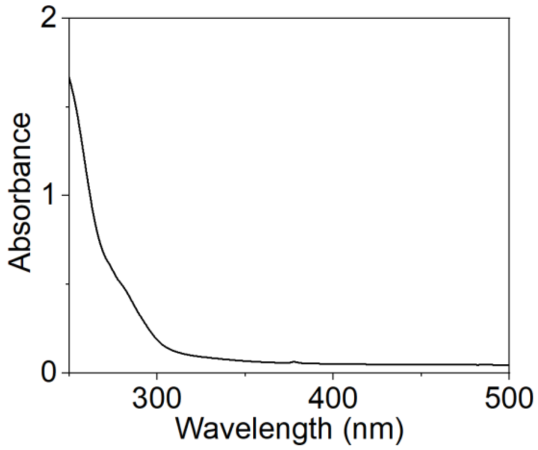


Supplementary Fig. 25. UV-vis spectrum of the as-prepared Er-BNON hybrid in hexane.

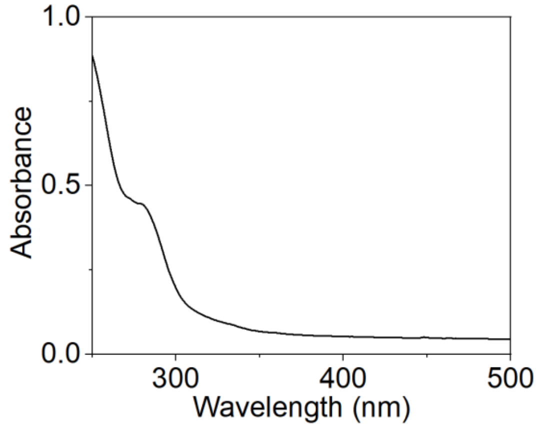


Supplementary Fig. 26. UV-vis spectrum of the as-prepared Ho-BNON hybrid in hexane.

## 4. Phosphorescence of ACA crystals and the Nd-ACA film

ACA crystals were prepared by a slow vapor-diffusion procedure. 3 mg/mL of a recrystallized ACA THF solution in a small vial was put into a large vial containing 5 mL of hexane and then sealed. The vials were kept steady in dark for 5 days, yielding bulky crystals that were further filtered and washed with hexane for further photophysical tests.

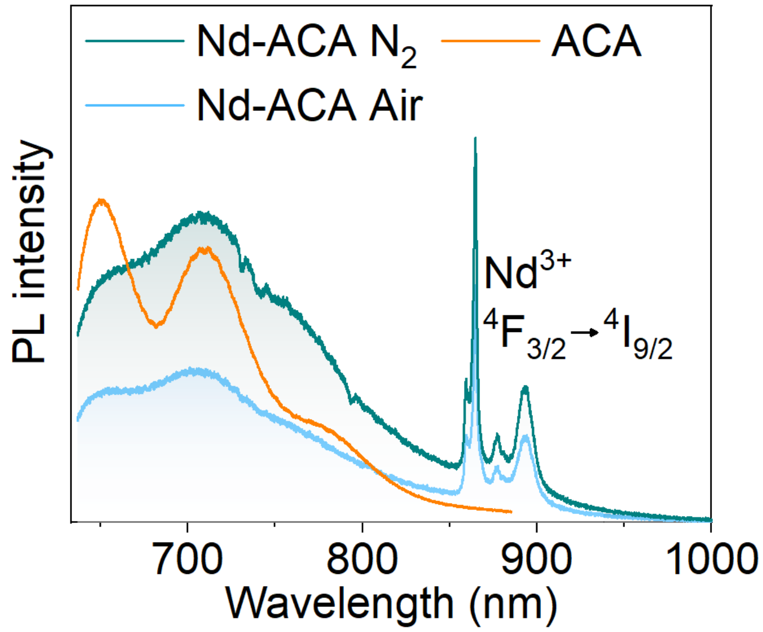


Supplementary Fig. 27. PL spectra of ACA crystals and the Nd-ACA film. The excitation wavelength was 633 nm. PL of ACA crystals was measured under ambient conditions. The Nd-ACA film was prepared by dropcasting a Nd-ACA hexane solution on a glass cover. The PL was firstly measured under ambient conditions (denoted as Nd-ACA Air, blue line). Then, the film was sealed in the glovebox by a quartz plate and UV-curing epoxy glue. The PL was then measured through the quartz plate (denoted as Nd-ACA $N_2$, green line).

## 5. TCSPC data

To measure the decay profile of a specific range of a PL spectrum, a long-pass filter and a short-pass filter were equipped.

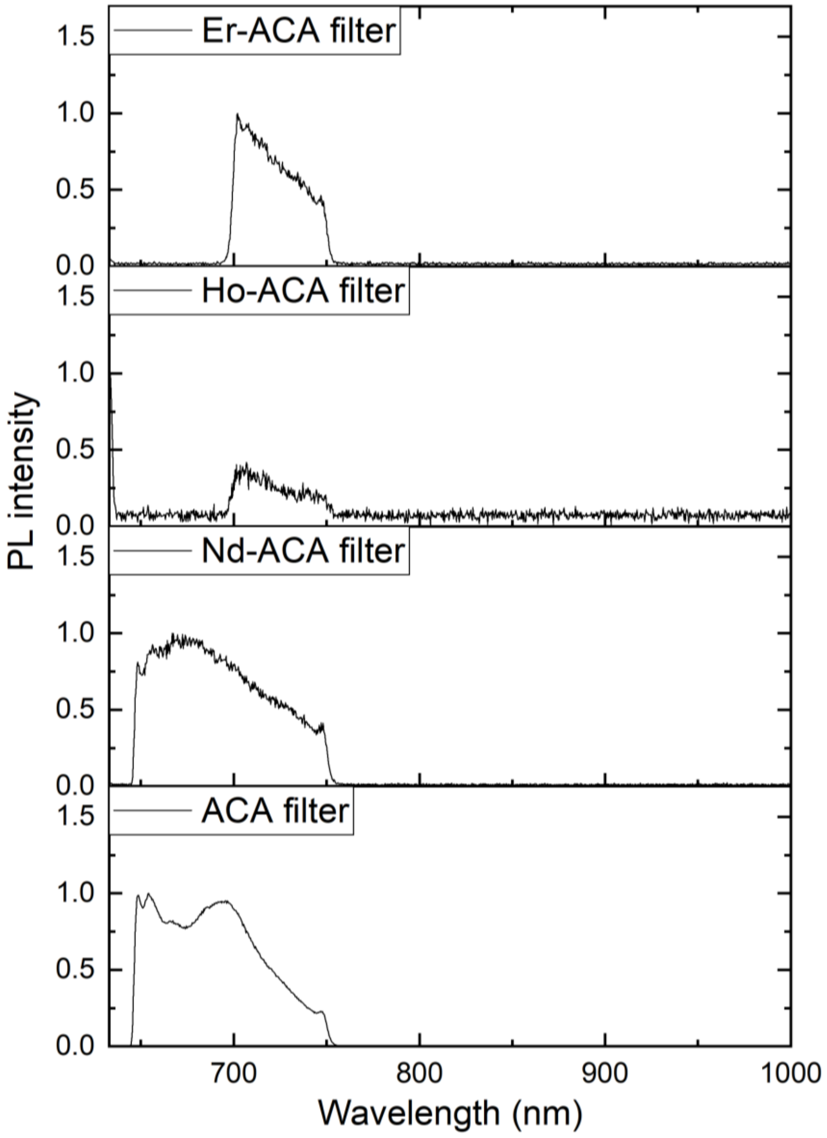


Supplementary Fig. 28. Partial PL spectra of ACA and Ln-ACA hybrids equipped with the long-pass filter and the short-pass filter for phosphorescence lifetime measurements. Emissions from $Ln^{3+}$ are eliminated by filters.

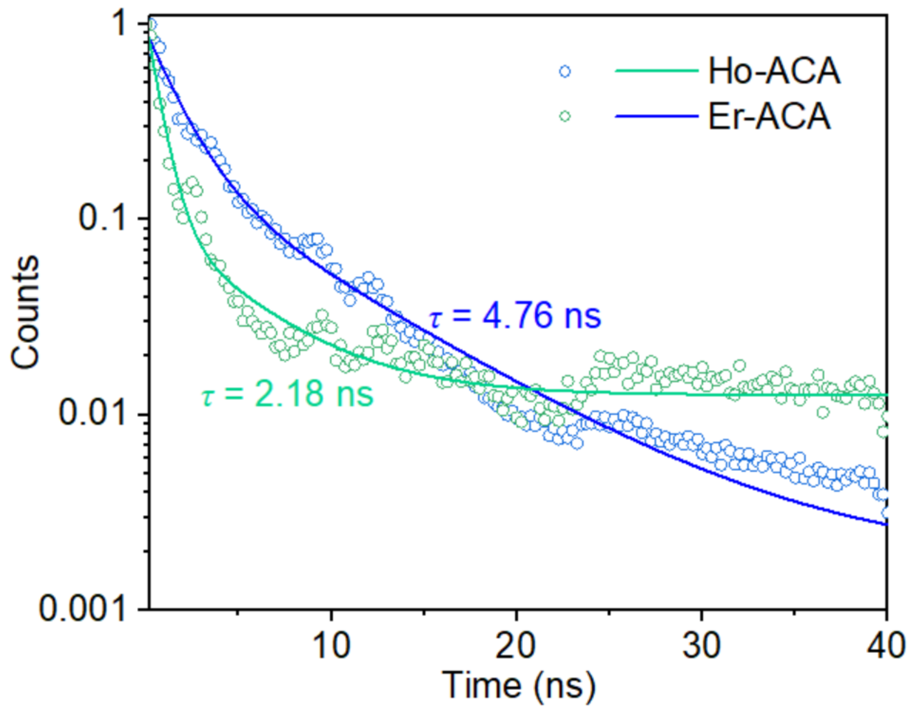


Supplementary Fig. 29. Decay profiles of ACA phosphorescence of Ho-ACA and Er-ACA hybrids.

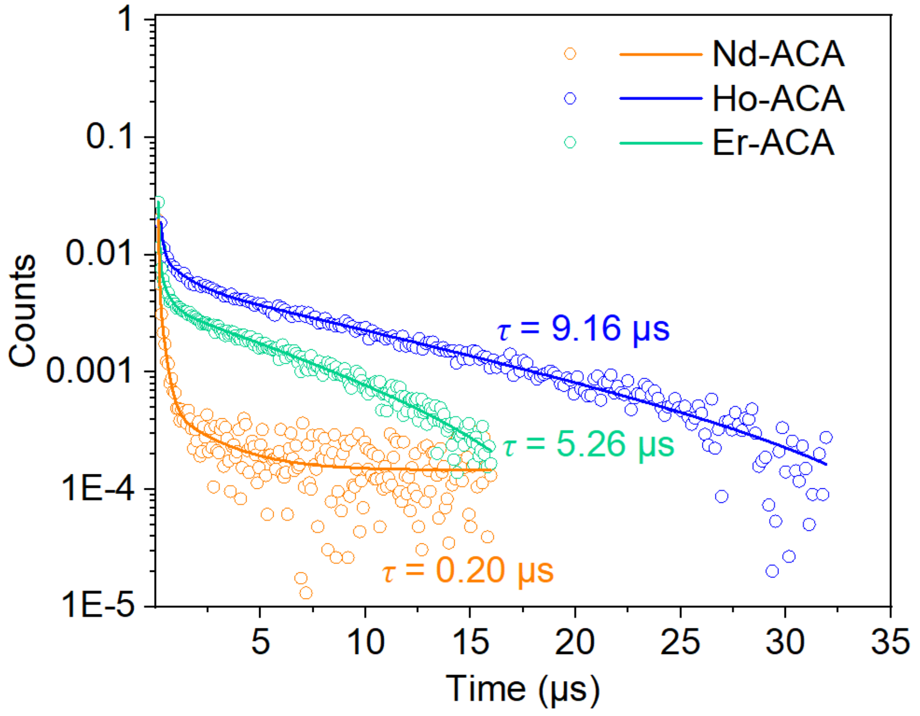


Supplementary Fig. 30. Decay profiles of lanthanide emissions of Nd-ACA, Ho-ACA and Er-ACA hybrids. A long-pass filter is equipped to remove most ACA phosphorescence. While due

to overlap between ACA phosphorescence and lanthanide emissions, each emission decay contains a fast component and a slow component. The fast one is ascribed to ACA phosphorescence and the slow one belongs to the lanthanide emission.

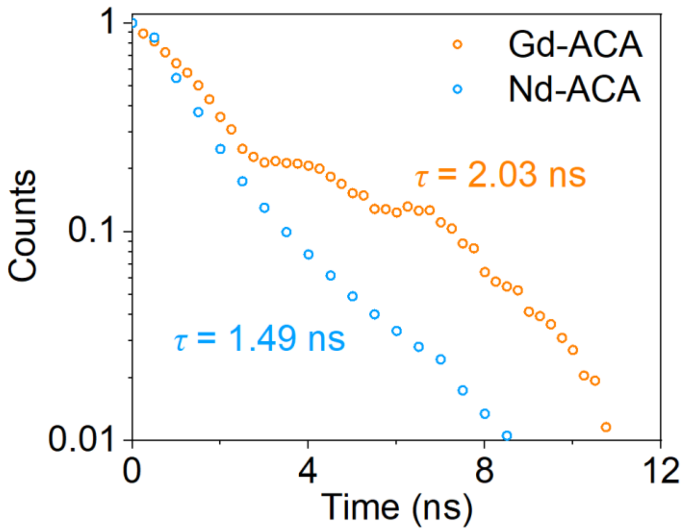


Supplementary Fig. 31. Comparison of emission decay of Gd-ACA (orange dots) and Nd-ACA (blue dots) at 650-750 nm in solution upon pulsed 640 nm excitation.

Table 1. Comparison of triplet lifetimes in different hybrid systems

| **Hybrid system** | **Lifetime (ns)** | **Explanation** |
|---|---|---|
| Gd-ACA | 2.03 | Compared with pure organic systems, the triplet lifetime is **shortened by spin-exchange coupling** that makes triplet emission no longer 'spin-forbidden'. |
| Nd-ACA | 1.49 | Compared with Gd-ACA, the triplet lifetime is **further shortened by triplet energy transfer**. |
| Nd@Y-ACA | 1.81 | Compared with Nd-ACA, the triplet lifetime is **prolonged by the inert Y shell** that weakened spin-exchange coupling and triplet energy transfer. |

## 6. Transient absorption data

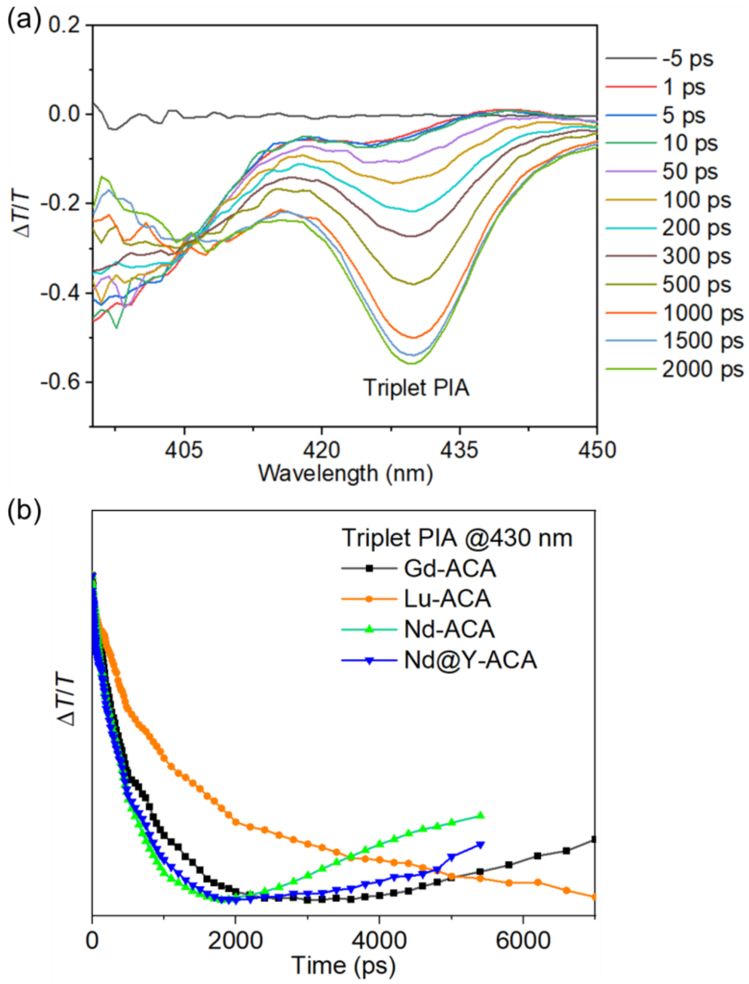


Supplementary Fig. 32. (a) Picosecond excited state dynamics of Nd-ACA in hexane measured by TA spectroscopy under excitation of 375 nm (28.6 μJ/cm$^2$) to show a rapid triplet rise. (b) Normalized kinetics extracted from ACA triplet PIA at 430 nm in Ln-ACA hybrids. Our maximum pump-probe delay is around 7000 ps. For triplet rise, Lu-ACA is the slowest on account of the lack of unpaired 4*f* electrons. The triplet in the Nd-ACA hybrid decays faster than that in the Nd@Y-ACA hybrid because the inert Y shell partially hampers triplet energy transfer to $Nd^{3+}$, and the triplet in the Gd-ACA hybrid decays the most slowly due to the lack of triplet energy transfer.

The triplet lifetime of ACA on the surface of the LnNCs is determined by both intrinsic decay and energy transfer from the ACA triplet to lanthanide ions. Lu-ACA has the slowest triplet rise and longest triplet lifetime because $Lu^{3+}$ lacks either unpaired 4*f* electrons or suitable energy levels, preventing both spin-exchange coupling and energy transfer. For Gd-ACA, Nd-

ACA and Nd@Y-ACA, their triplets rise rapidly at a similar rate and decay faster than that of Lu-ACA owing to unpaired 4*f* electron-induced spin-exchange coupling. Note that Nd-ACA refers to ACA coated on $NaGdF_4$:20%Nd. $Nd^{3+}$ is only doped in $NaGdF_4$, which contained 80% of $Gd^{3+}$. While in Lu-ACA, $Lu^{3+}$ is 100%.

Moreover, their triplet decays are also accelerated by energy transfer. As $Gd^{3+}$ doesn't have any energy level for triplet energy transfer but $Nd^{3+}$ has the $^4F_{3/2}$ energy level to accept triplet energy, Gd-ACA triplet decays slower than that of Nd-ACA. This is also consistent with the phosphorescence lifetimes for Gd-ACA and Nd-ACA, which are 2.03 ns and 1.49 ns, respectively. When Y shell is applied, the triplet energy transfer to $^4F_{3/2}$ and spin-exchange coupling are partially blocked, and thus on TA spectra, the Nd@Y-ACA triplet decays slower than Nd-ACA triplet but faster than Gd-ACA triplet. This phenomenon is also in line with the trend of phosphorescence lifetimes, which is Nd-ACA (1.49 ns) < Nd@Y-ACA (1.81 ns) < Gd-ACA (2.03 ns).

Overall, $Lu^{3+}$ doesn't have unpaired 4f electrons, so Lu-ACA has the longest triplet lifetime. Gd-ACA has a shorter triplet lifetime, as unpaired 4f electrons induce spin-exchange coupling. Nd-ACA exhibits the fastest triplet decay on account of both spin-exchange coupling and triplet energy transfer. Therefore, the TA data match well with TCSPC results.

## 7. Power-dependent emissions

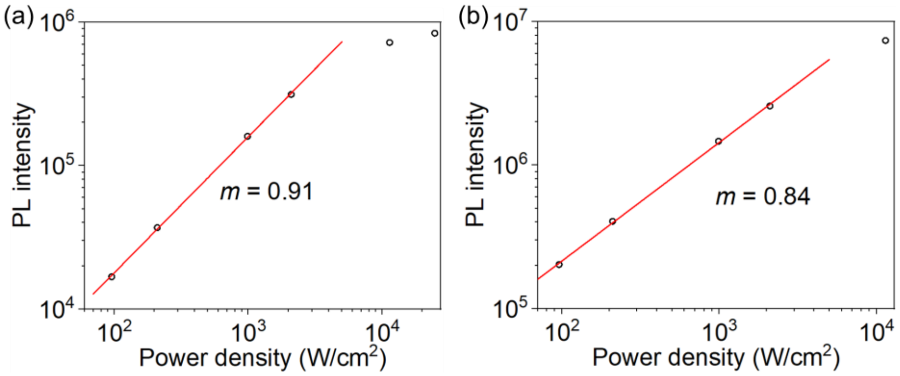


Supplementary Fig. 33. Power-dependent phosphorescence intensity of (a) Gd-ACA and (b) Nd-ACA. The concentration is 25 mg/mL. The excitation wavelength is 633 nm. Slope value indicates the exponent of a fitted power law: $I = A \cdot P^m$, where $I$ is the PL intensity, $A$ is the pre-exponent factor, $P$ is the incident power density, and $m$ is the slope or exponent close to 1.

Both Nd-ACA and Gd-ACA hybrids give linear power dependence, ruling out the possibility of triplet-triplet annihilation or two-photon absorption to generate a singlet exciton. The minimum power density to measure the phosphorescence is around 100 W/$cm^2$, which is lower than the common threshold for two-photon absorption upconverting that requires extremely high-power femtosecond lasers[5].

## 8. Surface loading calculation

The number of surface-bound ACA per single Gd nanocrystal is calculated based on following equations:

The volume of a single Gd nanocrystal ($V_{NC}$) is

$$V_{\mathrm{NC}} = \frac{4}{3} \times \left(\frac{d}{2}\right)^3 \times \pi = 179.5\ \mathrm{nm}^3$$

Where $d$ is the average diameter of Gd nanocrystals.

The mass of a single Gd nanocrystal ($m_{NC}$) is

$$m_{\mathrm{NC}} = \rho \times V_{\mathrm{NC}} = 1.1 \times 10^{-18}\ \mathrm{g}$$

Where $\rho$ is density of the nanocrystal. The density of hexagonal-phase $NaGdF_4$:25%Lu crystal is 5.85 g/cm$^3$.

It's difficult to precisely determine the mass fraction of OA in the OA-coated nanocrystal. The mass fraction of OA was 20%[6]. Therefore, the mass of a single OA-coated nanocrystal ($m_{NC\text{-}OA}$) is

$$m_{\mathrm{NC-OA}} = m_{\mathrm{NC}} \div 80\% = 1.4 \times 10^{-18}\ \mathrm{g}$$

The molecular weight of a single OA-coated nanocrystal ($M_{NC\text{-}OA}$) is

$$M_{\mathrm{NC-OA}} = m_{\mathrm{NC-OA}} \times N_A = 8.3 \times 10^5\ \mathrm{g/mol}$$

Where $N_A$ is the Avogadro constant, $6.02 \times 10^{23}$ mol$^{-1}$.

We added 1 mL of the 25 mg/mL nanocrystal stock solution for ligand exchange, and thus 25 mg of nanocrystals were used. The mole of nanocrystals used ($n_{NC\text{-}OA}$) is

$$n_{\mathrm{NC-OA}} = 25\ \mathrm{mg} \div M_{\mathrm{NC-OA}} = 3.0 \times 10^{-8}\ \mathrm{mol}$$

The concentration of ACA in Gd-ACA is determined by UV-vis. The absorbance of ACA (1 mg/mL) at 380 nm is 1.5. And the absorbance of 1 mL of Gd-ACA at 386 nm is 0.1. Therefore, the ACA in Gd-ACA is 0.06 mg, which equals to $2.7 \times 10^{-7}$ mol

The number of surface-bound ACA per single nanocrystal ($N_{\mathrm{ACA/NC}}$) is thus determined to be:

$$N_{\mathrm{ACA/NC}} = 2.7 \times 10^{-7} \div n_{\mathrm{NC-OA}} = 9 \text{ molecules}$$

The surface area of a single Gd nanocrystal ($A_{\mathrm{NC}}$) is

$$A_{\mathrm{NC}} = 4 \times \pi \times (\frac{d}{2})^2 = 154 \text{ nm}^2$$

The molecular area of ACA ($A_{\mathrm{ACA}}$) is estimated by regarding the molecule as a rectangle. The diameter of benzene (0.7 nm) is used to calculate the width (0.7 nm) and length (2.1 nm) of ACA. Therefore, $A_{\mathrm{ACA}} = 1.5$ nm$^2$. The maximum surface coverage ($C_{\mathrm{max}}$) is thus:

$$C_{max} = N_{\mathrm{ACA/NC}} \times A_{\mathrm{ACA}} \div A_{\mathrm{NC}} = 9\%$$

As ACA molecules have different conformations and orientations on the surface of the nanocrystal, the actual surface coverage is always less than $C_{\mathrm{max}}$.

The ACA molecules on the surface cover less than 9% of the surface area, which means the aromatic stacking of ACA or other chromophores is highly unlikely, i.e., we don't need to consider aggregation-caused quenching or excimer formation. Therefore, although chromophore loading can influence emission by aggregation, in our system, these chromophores are still well-separated on the surface.

When it comes to Pyrene and BNON chromophores and other lanthanide-doped nanocrystals, the situation is the same, as reflected by small absorbance of these chromophores on the surface and close nanocrystal diameters. Therefore, both the number of surface-bound chromophores and surface coverage is nearly consistent. The change in lifetime and emission intensity among these hybrids does not originate from different chromophore loading.

## 9. Effects of spin-orbital and spin-exchange couplings

Table 2. Comparison of spin-orbital and spin-exchange couplings

| **Mechanism** | **Triplet generation** | **Influenced by** |
|---|---|---|
| Spin-orbital coupling | Accelerating intersystem crossing ($S_1 \rightarrow T_1$) | 1. Heavy-atom effect, which is $\propto Z^4$<br>2. Structural effect |
| Spin-exchange coupling | Not only accelerating intersystem crossing ($S_1 \rightarrow T_1$) but also promoting direct triplet excitation from ground state ($S_0 \rightarrow T_1$) | 1. Unpaired electrons of lanthanide ions<br>2. Distance between lanthanide ions and organic chromophores |

Overall, the emission enhancement observed must be a combination of **(1)** the spin-orbit coupling (heavy atom effect, $\propto Z^4$) enhancement, and **(2)** the spin-exchange coupling enhancement. Both are in play, the spin-exchange enhancement is dominant in all cases considered, as $Gd^{3+}$ nanocrystals with 7 unpaired electrons have stronger phosphorescence enhancement than $Lu^{3+}$. In the case of ACA and BNON, the heavy atom effect seems to be negligible, however such a contribution is non-negligible for pyrene, resulting in even $Lu^{3+}$ enhancing the pyrene phosphorescence. This could be due to any of the factors controlling heavy-atom spin-orbit enhancement, including proximity of chromophore to the lanthanide ion, binding geometry, orbital overlaps, etc. Studying the complex interplay between heavy atom effect and spin-exchange and its impact on emission spectra requires detailed time-dependent density functional theory is being actively pursued, however it is outside the scope of this report of the initial discovery.

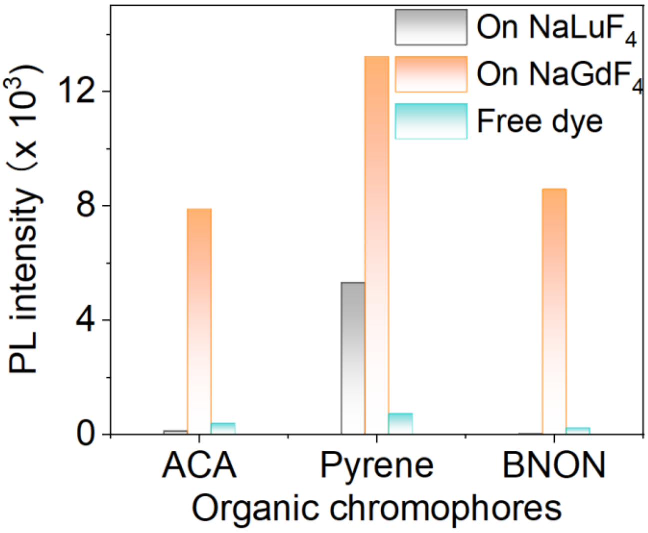


Supplementary Fig. 34. PL intensities of lanthanide-organic hybrids and free organic chromophores. The grey bar, organic bar and blue bar represent emission intensities of chromophores on the surface of $NaLuF_4$, $NaGdF_4$ and free dyes, respectively. The PL intensity is normalized according to the absorption of the surface-attached chromophore. We would also like to clarify that the $Lu^{3+}$ system **only** shows an emission enhancement for the pyrene system, and not for any other chromophore we have considered and still significantly lower than the Gd-induced spin-exchange coupling phosphorescence. Additionally, the emission from pyrene is more complex, and may involve the formation of transient excimers with free-floating molecules in solution as the surface coordination is reversible.

## 10. Phosphorescence quantum yield ($\Phi_P$) and molar absorption coefficient ($\varepsilon$) of Gd-ACA in solution

### Lower-bound estimation of $\Phi_P$

To calculate the triplet's photoluminescence quantum yield (PLQY), we measured the luminescence of the solution phase samples with controlled absorbance and concentrations under the microscope to collect more emitted light. A conventional method of measuring a reference molecule with a known and easily measured PLQY was then used to calibrate the microscope's collection efficiency and hence obtain the PLQY of the desired system. We used the best practices detailed in[7,8], such as a good overlap between the absorption-emission spectrum of the reference compound, identical solvents and nominal concentrations, identical excitation-collection geometries in the microscope setup, and identical excitation wavelengths.

Our reference compound used was 6,13-bis(triisopropylsilylethynyl)pentacene-2-carboxylic acid (TipsPC). The quantum yield of this reference compound ($\Phi_{Ref}$) was determined on an Edinburgh FLS1000 spectrometer equipped with the integral sphere under excitation of 633 nm using Eqn.

$$\Phi_{\mathrm{Ref}} = \frac{Number\ of\ photons\ emitted}{Number\ of\ photons\ absorbed}$$

According to previous published methods to measure absolute quantum yield[9], three individual experiments were carried out, including measuring the spectrum when the sphere was empty, measuring the emission when the laser was directed onto the sphere wall in the presence of TipsPC and measuring the emission when the laser was directed onto TipsPC. $\Phi_{Ref}$(TipsPC) was thus determined to be 15.3%.

$\Phi_P$ of Gd-ACA was measured via a relative quantum yield procedure using Eqn. (1)

$$\Phi_{\mathrm{P}} = \Phi_{\mathrm{Ref}} \times \left(\frac{I_{\mathrm{P}}}{I_{\mathrm{Ref}}}\right) \times \left(\frac{1 - 10^{-A_{\mathrm{Ref}}}}{1 - 10^{-A_{\mathrm{P}}}}\right) \times \left(\frac{n_{\mathrm{P}}}{n_{\mathrm{Ref}}}\right)^2 \qquad \text{Eqn. (1)}$$

where the subscripts P and R denote Gd-ACA and TipsPC reference, respectively. $I$ is the integrated PL spectrum intensity upon excitation with 633 nm under the microscope. $A$ is the absorbance of the solution at 633 nm, and $n$ is the refractive index of the solution solvent.

The collected PL intensity of the reference and the desired compounds are shown in Supplementary Fig. 35, normalized for excitation power and integration time of the spectrometer. We can see a good spectral overlap between the emission peaks, which we integrate in their entirety to estimate $I_P$=14611.9 and $I_{Ref}$=13342135.6.

Supplementary Fig. 35shows the absorbance of the TipsPc sample $A_{Ref}$=0.027 is reliably measured due to the singlet absorption bands being strong, however the triplet absorption of Gd-ACA is more difficult to measure, due to the lower oscillator strength. Increasing this signal is possible but only in the solid state as a film, which makes comparison to solution measurements challenging. Hence, measurement of the absorbance spectrum in a cuvette of the Gd-ACA nanocrystals, at concentrations before sedimentation, yield the upper bound of the absorbance $A_P$=0.00016 of the Gd-ACA triplet.

Since the absorbance $A_P$ is an upper-bound and appears in the denominator of Eqn. (1), the estimated $\Phi_P$(Gd-ACA) is thus a lower-bound, and our actual PLQY must be greater than the value calculated here, which is $\Phi_P$(Gd-ACA) = 2.7%.

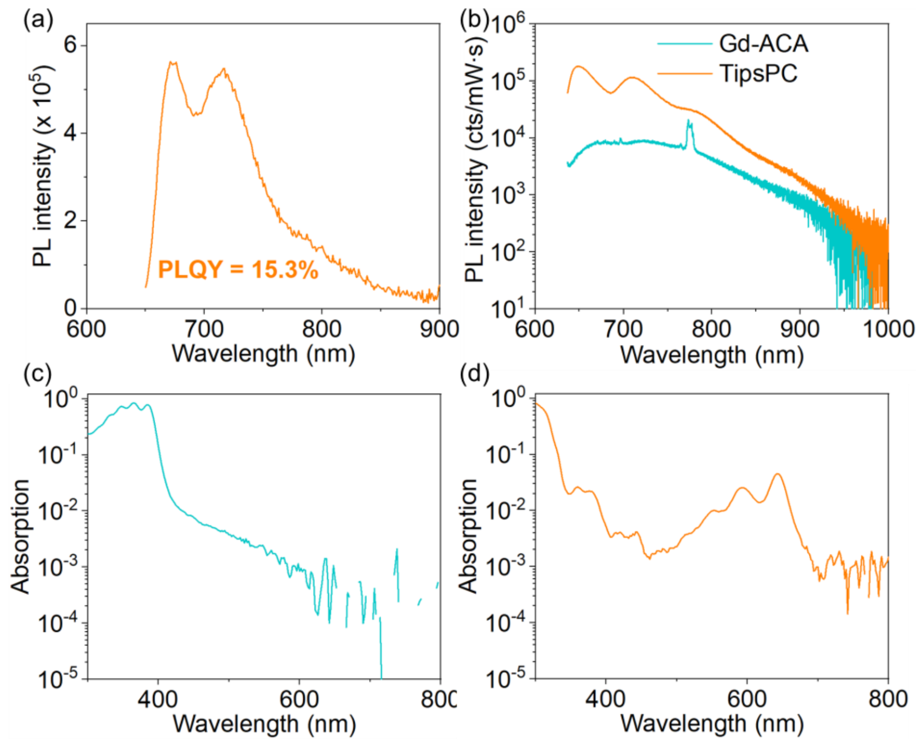


Supplementary Fig. 35. Measurement of phosphorescence quantum yield. (a) PL spectrum and absolute quantum yield of TipsPC under excitation of 633 nm in solution under ambient

conditions. (b) Normalized PL spectra of Gd-ACA and TipsPC under excitation of 633 nm in solution under ambient conditions. Absorption spectra of (c) Gd-ACA and (d) TipsPC in hexane.

### Upper-bound estimation of $\varepsilon$

We may place an upper-bound on such a molar absorption coefficient of the triplet, which is 53.3 $M^{-1}cm^{-1}$. This number is calculated based on the equation

$$A_{\mathrm{P}} = \varepsilon \times c_{\mathrm{NC}} \times l$$

where $A_{\mathrm{P}}$ is the absorbance of Gd-ACA during $\Phi_{\mathrm{P}}$ measurements, which is 0.00016. $\varepsilon$ is the molar absorption coefficient. $c_{\mathrm{NC}}$ is the concentration of Gd-ACA, which is $3 \times 10^{-5}$ M (has been calculated earlier). $l$ is the light pass length during absorbance measurement, which is 0.1 cm.

## 11. Cryogenic frozen solution phosphorescence measurements

We measured phosphorescence of the ACA solution under cryogenic temperatures. ACA hexane solution was first frozen by liquid nitrogen and then put under the microscope immediately for emission measurement. The orange spectrum shown in Supplementary Fig. 36 was taken rapidly when hexane was still frozen, and thus we infer the temperature of the solution is below 177 K. After keeping the frozen solution under room temperature for minutes, the solution melted, and its emission was taken again, affording the black spectrum in Supplementary Fig. 36.

By comparison, we observe that both Nd-ACA and ACA solution below 177 K show identical vibronic signals at around 700 nm, indicating their emission originate from the same excited state. During hexane melting and temperature rising, the emission of ACA solution faded rapidly, resulting in negligible emission at around 700 nm. We didn't observe any emission at room temperature. The temperature-dependent emission at around 700 nm of ACA solution implies that the emission is from the ACA triplet. Therefore, the comparison demonstrates that the Ln-ACA emission at 700 nm is indeed phosphorescence.

Additionally, by normalizing emission spectra to the Raman signal of hexane, the phosphorescence intensity of the Nd-ACA solution at room temperature is higher than that of the ACA solution at cryogenic temperature, indicating that our strategy to brighten the triplet is more efficient and useful.

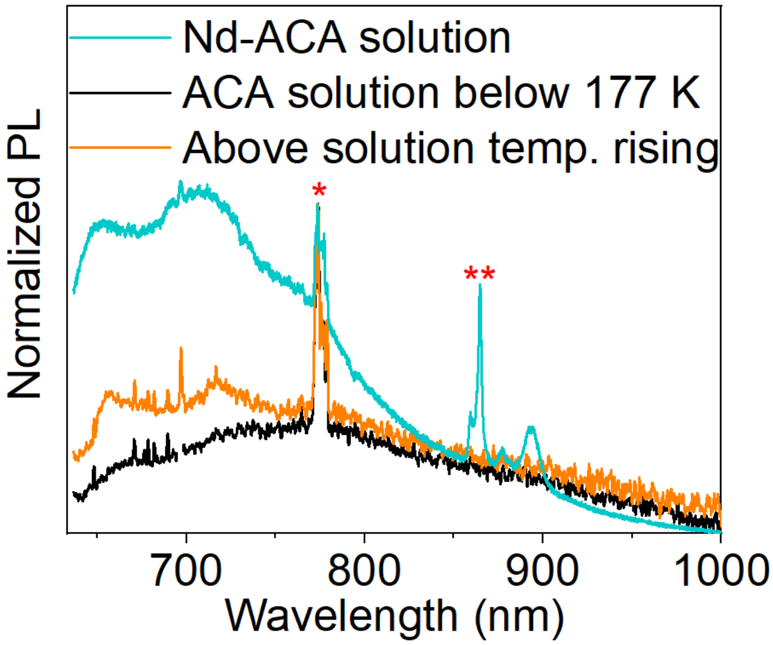

Supplementary Fig. 36. Comparison between phosphorescence spectrum of Nd-ACA (cyan line), and ACA hexane solution (1 mg/mL) during the liquid nitrogen-frozen process (orange line) and melting process (black line) under excitation with 633 nm. The emission intensities are normalized with the Raman peak of hexane (marked with asterisk). $Nd^{3+}$ emission is marked with double asterisks.

## 12. Energy diagram

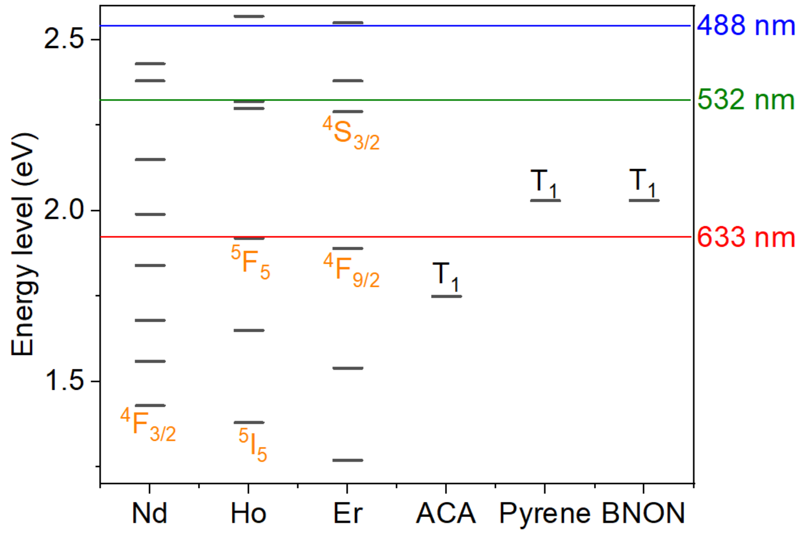


Supplementary Fig. 37. Energy diagram of lanthanide ions and organic chromophores. The lasers used to excite triplets are shown in blue, green and red, respectively. The emissive energy levels of lanthanide ions are marked.

By investigating different organic chromophores, we also demonstrate that this method works for visible and NIR dyes. Although we infer that those triplets in chromophores that do not have coordination groups (e.g. a carboxylic acid group) may not be efficiently brightened as those chromophores cannot be attached on the surface of LnNCs. This could be a potential limitation. One possible solution is to remove the OA ligands on the LnNCs and then blend them with organic chromophores to form a film. The distance between LnNCs and organics may thus be shortened in the solid state to induce efficient spin-exchange coupling.

## 13. Wavelength-dependent emission spectra

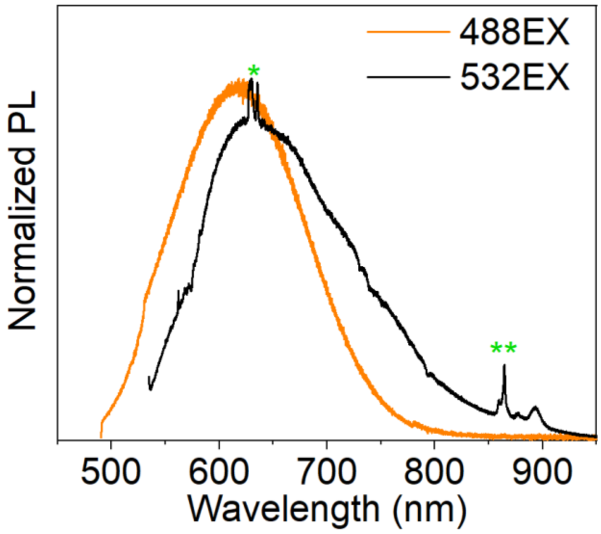


Supplementary Fig. 38. Normalized PL spectra of the Nd-BNON solution under 488 nm (red line) and 532 nm (black line) excitation. The Raman peak of hexane under 532 nm excitation is marked with asterisk, and $Nd^{3+}$ emission is marked with double asterisks.

The system does not have excitation wavelength dependence. The earlier analysis shows that exciting the Nd-BNON hybrid with either 532 nm or 488 nm results in very similar emission maxima. With 488 nm, the high-lying BNON triplet is more efficiently excited than with 532 nm, resulting in the 488 nm-excited triplet emission being much stronger than the hexane Raman peak, thus suppressing its magnitude relative to the 532 nm-excited spectrum.

## 14. Computational studies of spin-exchange coupling

### Computational methods

The geometries of the [Gd-ACA]$^{2+}$ and ACA$^{1-}$ alone complexes were optimized using the Gaussian 16 software[10], employing Unrestricted Density Functional Theory (U-DFT) with the CAM-B3LYP long-range corrected exchange-correlation functional[11]. The Gd atom was treated with the Stuttgart/Dresden (SDD)[12,13] basis set and effective core potential (ECP), while the def2-SVP[14] basis set was used for all other atoms. Grimme's D3 dispersion correction with Becke–Johnson damping[15] was applied to account for van der Waals interactions, together with a fine integration grid. Frequency calculations were performed on the final geometry to confirm that the optimized structure corresponds to a true energy minimum. Given that the systems are small and relatively simple, this level of theory is considered sufficient to accurately predict the geometry of the ground state.

We next employed a Complete Active Space Self-Consistent Field (CASSCF) multiconfigurational approach[16] to describe excited state properties of both systems. This method has access to all relevant configuration state functions (CSFs) and therefore maintains spin-purity, besides capturing crucial static electron correlation effects. Based on the multiconfigurational wavefunction, dynamic electron correlation effects were subsequently recovered with second-order perturbation theory correction. To do this, we utilized the N-Electron Valence State Perturbation Theory (NEVPT2) method developed by Angeli and co-workers[17,18], which recovers missing dynamic correlation while remaining intruder-state free through the implementation of Dyall's Hamiltonian[19]. This feature helps prevent divergence when electronic states are nearly degenerate. In all calculations, we employed the multi-state quasi-degenerate NEVPT2 (QD-NEVPT2) formulation[20]. This method constructs an effective Hamiltonian in the space of the selected CASSCF states and then diagonalize it to introduce coupling between states, thereby avoiding non-physical artificial state crossings.

All CASSCF+QD-NEVPT2 calculations were performed using the ORCA 6.1 software[21–25]. We began with a single-point U-DFT calculation (employing the CAM-B3LYP exchange–correlation functional) to generate Quasi-Restricted molecular Orbitals (QROs), which have been demonstrated to be an optimal starting point for CASSCF calculations in open shell systems[26]. For this step and the subsequent CASSCF, the Zeroth-Order Regular Approximation

(ZORA)[27] was employed to include relativistic corrections. The SARC2-ZORA-QZV[28] basis set was used for the Gd, while the ZORA-def2-SVP basis set was used for all other atoms. These basis sets are parameterized for accurate performance with the ZORA correction. Moreover, the use of a quadruple-zeta basis set for Gd atom is recommended because it exhibits strong correlation effects, which require a flexible basis set for proper description, while still maintaining the computational feasibility of the CASSCF+QD-NEVPT2 calculation. The Atomic Valence Active Space (AVAS)[29] approach was utilized to obtain the Gd valence f-orbitals, providing an optimal starting guess for the subsequent CASSCF calculation. By using QRO orbitals as an initial guess, a preliminary CASSCF calculation was performed to verify orbitals selection prior to the full QD-NEVPT2 calculation. We then gradually expanded the active space CAS(nr. of active $e^-$, nr. of active orbitals) to include as many relevant orbitals as possible, while maintaining HOMO/LUMO symmetry and balancing the number of orbitals localized in different regions of the system[30,31] We determined that accurate results for $[Gd\text{-}ACA]^{2+}$ could be obtained using a CAS(13,13) active space. This space comprised the HOMO, HOMO-1, and HOMO-2 of the $ACA^{-1}$ and their corresponding three LUMO orbitals, along with the seven Gd 4f-orbitals. Similarly, we selected a CAS(6,6) for the isolated $ACA^{-1}$ considering the same 3 HOMOs and correspondent LUMOs. Plots of these orbitals are provided in the Supporting Information Section 14. Further expansion of the active space was computationally not feasible or not bringing any relevant change in the results. We employed a state-averaged approach averaging over the low-lying 7 octuplet states (S = 7/2) and 3 decuplet states (S = 9/2). We were particularly interested in the states arising from the coupling between the $ACA^{-1}$ and the Gd f-orbitals. The first Gd excited state ($^6P_{7/2}$) lies at a significantly higher energy and is therefore not expected to be involved in the relevant system dynamics. Spin-orbit coupling (SOC) matrix elements between the calculated roots were computed for both the $[Gd\text{-}ACA]^{+2}$ complex and the isolated $ACA^{-1}$ employing an mean-field (SOMF) approximation to the full Breit-Pauli spin-orbit Hamiltonian operator[32].

## Computational results

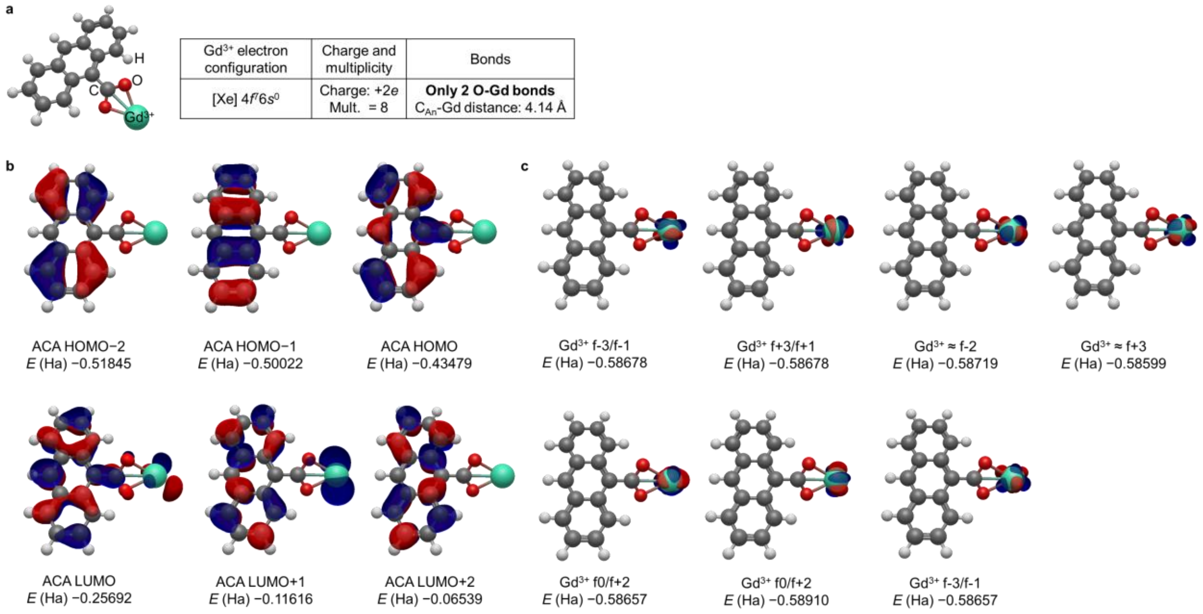


Supplementary Fig. 39. Orbitals of $Gd^{3+}$-ACA complex. **a**. The complex structure used for calculation. **b** and **c**. Active space orbitals for the complex. **Note that there is a partial mixing between LUMO and LUMO+1 with $Gd^{3+}$ orbitals.** An isovalue of 0.03 e/au$^3$ was used to plot the orbitals.

a

| | CAS + QD-NEVPT2 | Nature | Experiment |
|---|---|---|---|
| $D_1$ (eV) Mult. 10 | 1.904 | 92% H→L [22uuuuuuuuu00] | No Data |
| *f* | (SOC) $2.6e^{-7}$ | | |
| $O_1$ (eV) Mult. 8 | **1.941** | 40% (0.633) [22duuuuuuuu00] 32% (0.563) [22uuuuuuuud00] | 1.83 (Emission) |
| *f* | **$1.4e^{-4}$ ($\varepsilon_I^{max} \approx 5.45\ M^{-1}cm^{-1}$)** | **20% 7 configurations with around 3% contributions: [22uuuuuuudu00], [22uuuuuuduu00]...** | **Triplet** |
| $O_2$ (eV) Mult. 8 | 2.353 | 41% (0.640) [22duuuuuuuu00] 49% (-0.699) [22uuuuuuuud00] | 3.1 (Absorption) |
| *f* | $9.0e^{-2}$ | $4.9e^{-2}$ % 7 configurations: [22uuuuuuduu00],... | Singlet |

b

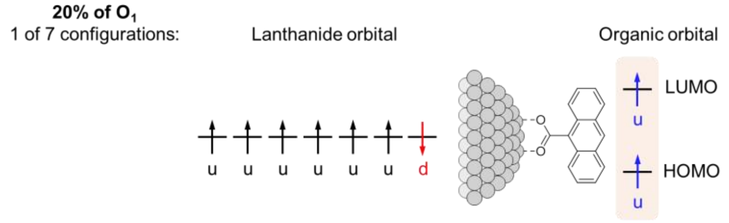


c

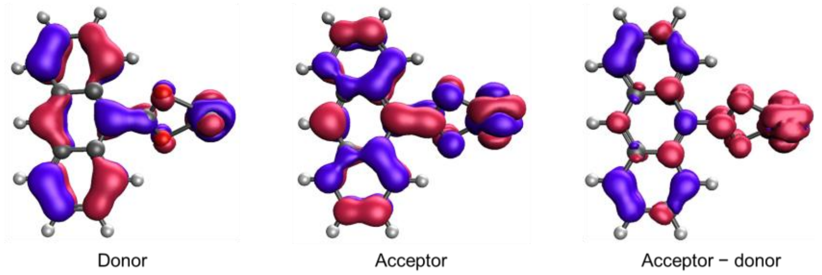


Supplementary Fig. 40. **a**. Tables of energy levels, oscillator strengths and transitions obtained from CASSCF+QD-NEVPT2 for $Gd^{3+}$-ACA complex. The last column includes experimental results obtained in this work. In CASSCF, the electronic states are described as a linear combination of individual electronic configurations, which are formally represented here by a 13-digit sequence enclosed in brackets (e.g., [22uu...]). Each position in the sequence corresponds to a specific molecular orbital in the active space, ordered as: 3 ACA-HOMOs, 7 f-orbitals of Gd, and 3 ACA-LUMOs. '2' indicates a double occupied orbital, 'u' indicates a 'spin up' electron, 'd' indicates a 'spin down' electron and 0 an empty orbital. For example, in the ferromagnetic decuplet state $D_1$ the dominant configuration's blue labels u corresponds to spin in of the HOMO and LUMO of ACA while the black u labels corresponds to the spin up in the 4f orbitals of Gd(III). The reported oscillator strength of the $D_1$ state includes spin-orbit coupling effects. **b**. Schematic illustration of 1 of 7 configurations of the 20% of $O_1$ state in which one of the f-orbitals' electron is flipped down. **c**. Highest occupied and lowest unoccupied natural transition orbitals for the triplet-like state $O_1$. Our results show a

contribution of around 8% and 3% of f orbitals in the donor and acceptor densities, respectively. However, a reorganization of the f-orbitals electron is also involved in the state. This shows that the $O_1$-triplet like state transition is not coming only from a HOMO to LUMO transition but also involves the f-orbitals of $Gd^{3+}$, which contributes to the $O_1$ oscillator strength.

The theoretical investigation of the excited states of the system focused on the three low-lying states corresponding to various spin combinations possible for the hybrid molecular-lanthanide system. The lowest energy state is the "ferromagnetic" state where all spin states on the organic and lanthanide are aligned ($D_1$ – indicating a decuplet with 9 unpaired electrons $S = 9/2$), which cannot be excited optically due to the spin-forbidden character of the transition and the weak spin-orbit coupling (spin-orbit coupling term $SOC_{O0-D1}$=0.101 $cm^{-1}$). The next state is our key luminescent triplet-like state $O_1$ (O indicating octet of 7 unpaired spins $S$ = 7/2), which has a wavefunction composed of a superposition of 72% of the $Ms$ = 0 triplet state, and 20% of the $Ms$ = ±1 state where the overall octet is preserved via a spin flip on the lanthanide 4f orbital. A calculation of the donor and acceptor Natural Transition Orbitals (NTOs) clarifies that the main orbitals involved in the $O_1$ transition are the HOMO and LUMO of ACA. Interestingly, both the Highest Occupied and Lowest Unoccupied NTOs, corresponding to the hole and particle, respectively have a significant component originating from the Gd f-orbitals. Such a difference is likely attributed to the fact that our calculation is based on a vertical transition and neglects solvent effects. The highest energy state is the overall molecular singlet-like state $O_2$ weakly coupling with the Gd. The energy of this state is red shifted compared to what was found experimentally but clearly well separated from the $O_1$ state so that there is no ambiguity on the origin of the emission.

From computational studies, we can confirm the following points: 1. There is a partial orbital overlap between ACA-LUMOs and $Gd^{3+}$ orbitals (Figure R1b), which makes direct spin-exchange coupling possible. 2. The spin-exchange coupling is effective upon direct photoexcitation of the triplet-like state $O_1$ (Figure R2a). This is reflected by a 20% contribution to the wavefunction of transitions involving two 'spin up' electrons in ACA HOMO and LUMO and one 'spin down' electron in one of the 4f orbitals. 3. The computed absorption coefficient of the direct triplet excitation is about 5.45 $M^{-1}cm^{-1}$, which is comparable (yet smaller) to the experimental value (upper estimate of ~50 $M^{-1}cm^{-1}$). In view of the simplicity of the model used (a simplified chemical structure, no vibronic Herzberg-Teller coupling, …), we consider this is a reasonable agreement. Most importantly, it supports the picture that the finite oscillator strength for the direct triplet absorption is induced by a double spin-flip mechanism (with one

flip on the ACA and counter flip from the unpaired 4f electrons of $Gd^{3+}$), which results in an intensity borrowing from the optically-allowed singlet. We expect this intensity borrowing to be more efficient for dyes with reduced singlet-triplet exchange energy, a strategy we are currently exploring. The spin-orbit coupling computed between the $O_1$ and $O_0$ and states (associated with the transition from the $T_1$ to the $S_0$ of the ACA core) and $O_2$ and $O_1$ (corresponding to the coupling between $T_1$ and $S_1$ of the ACA core) show an increase by approximately eight times in presence of Gd (comparing the $ACA^{-1}$ alone to the $[Gd\text{-}ACA]^{+2}$ complex), which should thus speed up the singlet-to-triplet conversion compared to the pure ligand.

## 15. References


1. Utochnikova, V. V. *et al.* Lanthanide 9-anthracenate: solution processable emitters for efficient purely NIR emitting host-free OLEDs. *J. Mater. Chem. C* **4**, 9848-9855, (2016).

2. Han, S. *et al.* Lanthanide-doped inorganic nanoparticles turn molecular triplet excitons bright. *Nature* **587**, 594-599, (2020).

3. Jiang, Z. *et al.* Ultra-wideband-responsive photon conversion through co-sensitization in lanthanide nanocrystals. *Nat. Commun.* **14**, 827, (2023).

4. Jakob, L. A. *et al.* Single photon multiclock lock-in detection by picosecond timestamping. *Optica* **8**, 1646-1653, (2021).

5. Rumi, M. & Perry, J. W. Two-photon absorption: an overview of measurements and principles. *Adv. Opt. Photon.* **2**, 451-518 (2010).

6. Wei, W. *et al.* Alleviating Luminescence Concentration Quenching in Upconversion Nanoparticles through Organic Dye Sensitization. *J. Am. Chem. Soc.* **138**, 15130-15133, (2016).

7. Würth, C., Grabolle, M., Pauli, J. *et al.* Relative and absolute determination of fluorescence quantum yields of transparent samples. *Nat. Protoc.* **8**, 1535–1550 (2013).

8. Würth, C., Grabolle, M., Pauli, J., Spieles, M. & Resch-Genger, U. Comparison of Methods and Achievable Uncertainties for the Relative and Absolute Measurement of Photoluminescence Quantum Yields. *Anal. Chem.* **83**, 3431-3439, (2011).

9. de Mello, J. C., Wittmann, H. F. & Friend, R. H. An improved experimental determination of external photoluminescence quantum efficiency. *Adv. Mater.* **9**, 230-232, (1997).

10. Frisch, M. J.; Trucks, G. W.; Schlegel, H. B.; Scuseria, G. E.; Robb, M. A.; Cheeseman, J. R.; Scalmani, G.; Barone, V.; Petersson, G. A.; Nakatsuji, H.; Li, X.; Caricato, M.; Marenich, A. V.; Bloino, J.; Janesko, B. G.; Gomperts, R.; Mennucci, B.; Hratchian, H. P.; Ortiz, J. V.; Izmaylov, A. F.; Sonnenberg, J. L.; Williams-Young, D.; Ding, F.; Lipparini, F.; Egidi, F.; Goings, J.; Peng, B.; Petrone, A.; Henderson, T.; Ranasinghe, D.; Zakrzewski, V. G.; Gao, J.; Rega, N.; Zheng, G.; Liang, W.; Hada, M.; Ehara, M.; Toyota, K.; Fukuda, R.; Hasegawa, J.; Ishida, M.; Nakajima, T.; Honda, Y.; Kitao, O.; Nakai, H.; Vreven, T.; Throssell, K.; Montgomery, J. A., Jr.; Peralta, J. E.; Ogliaro, F.; Bearpark, M. J.; Heyd, J. J.; Brothers, E. N.; Kudin, K. N.; Staroverov, V. N.; Keith, T. A.; Kobayashi, R.; Normand, J.; Raghavachari, K.; Rendell, A. P.; Burant, J. C.; Iyengar, S. S.; Tomasi, J.; Cossi, M.; Millam, J. M.; Klene, M.;

Adamo, C.; Cammi, R.; Ochterski, J. W.; Martin, R. L.; Morokuma, K.; Farkas, O.; Foresman, J. B.; Fox, D. J. Gaussian 16 Revision A.03, 2016.

11. Yanai, T.; Tew, D. P.; Handy, N. C. A New Hybrid Exchange–Correlation Functional Using the Coulomb-Attenuating Method (CAM-B3LYP). *Chem. Phys. Lett.* **393**, 51–57, (2004).

12. Cao, X.; Dolg, M. Valence Basis Sets for Relativistic Energy-Consistent Small-Core Lanthanide Pseudopotentials. *J. Chem. Phys.* **115**, 7348–7355, (2001).

13. Cao, X.; Dolg, M. Segmented Contraction Scheme for Small-Core Lanthanide Pseudopotential Basis Sets. *J. Mol. Struct. THEOCHEM*, **581**, 139–147, (2002).

14. Weigend, F.; Ahlrichs, R. Balanced Basis Sets of Split Valence, Triple Zeta Valence and Quadruple Zeta Valence Quality for H to Rn: Design and Assessment of Accuracy. *Phys. Chem. Chem. Phys.* **7**, 3297, (2005).

15. Grimme, S.; Ehrlich, S.; Goerigk, L. Effect of the Damping Function in Dispersion Corrected Density Functional Theory. *J. Comput. Chem.* **32**, 1456–1465, (2011).

16. Lindh, R.; Galván, I. Fdez. Multi-Configurational Reference Perturbation Theory with a CASSCF Reference Function. In *Quantum Chemistry and Dynamics of Excited States*; González, L., Lindh, R., Eds.; Wiley, 2020; pp 299–353.

17. Schapiro, I.; Sivalingam, K.; Neese, F. Assessment of N-Electron Valence State Perturbation Theory for Vertical Excitation Energies. *J. Chem. Theory Comput.* **9**, 3567–3580, (2013).

18. Angeli, C.; Cimiraglia, R.; Evangelisti, S.; Leininger, T.; Malrieu, J.-P. Introduction of *n* - Electron Valence States for Multireference Perturbation Theory. *J. Chem. Phys.* **114**, 10252–10264, (2001).

19. Dyall, K. G. The Choice of a Zeroth-Order Hamiltonian for Second-Order Perturbation Theory with a Complete Active Space Self-Consistent-Field Reference Function. *J. Chem. Phys.* **102**, 4909–4918, (1995).

20. Angeli, C.; Borini, S.; Cestari, M.; Cimiraglia, R. A Quasidegenerate Formulation of the Second Order N-Electron Valence State Perturbation Theory Approach. *J. Chem. Phys.* **121**, 4043–4049, (2004).

21. Lang, L.; Sivalingam, K.; Neese, F. The Combination of Multipartitioning of the Hamiltonian with Canonical Van Vleck Perturbation Theory Leads to a Hermitian Variant of Quasidegenerate N-Electron Valence Perturbation Theory. *J. Chem. Phys.* **152**, 014109, (2020).

22. Neese, F. Software Update: The ORCA Program System—Version 6.0. *WIREs Comput. Mol. Sci.*, **15**, e70019, (2025).

23. Kollmar, C.; Sivalingam, K.; Helmich-Paris, B.; Angeli, C.; Neese, F. A Perturbation-based super-CI Approach for the Orbital Optimization of a CASSCF Wave Function. *J. Comput. Chem.* **40**, 1463–1470, (2019).

24. Guo, Y.; Sivalingam, K.; Neese, F. Approximations of Density Matrices in N-Electron Valence State Second-Order Perturbation Theory (NEVPT2). I. Revisiting the NEVPT2 Construction. *J. Chem. Phys.* **154**, 214111, (2021).

25. Ugandi, M.; Roemelt, M. A Recursive Formulation of One-electron Coupling Coefficients for Spin-adapted Configuration Interaction Calculations Featuring Many Unpaired Electrons. *Int. J. Quantum Chem.* **123**, e27045, (2023).

26. Neese, F. Importance of Direct Spin−Spin Coupling and Spin-Flip Excitations for the Zero-Field Splittings of Transition Metal Complexes: A Case Study. *J. Am. Chem. Soc.* **128**, 10213–10222, (2006).

27. Van Lenthe, E.; Baerends, E. J.; Snijders, J. G. Relativistic Total Energy Using Regular Approximations. *J. Chem. Phys.* **101**, 9783–9792, (1994).

28. Aravena, D.; Neese, F.; Pantazis, D. A. Improved Segmented All-Electron Relativistically Contracted Basis Sets for the Lanthanides. *J. Chem. Theory Comput.* **12**, 1148–1156, (2016).

29. Sayfutyarova, E. R.; Sun, Q.; Chan, G. K.-L.; Knizia, G. Automated Construction of Molecular Active Spaces from Atomic Valence Orbitals. *J. Chem. Theory Comput.* **13**, 4063–4078, (2017).

30. Roos, B. O. Multiconfigurational Quantum Mechanics ( QM ) for Heavy Element Compounds. In *Encyclopedia of Inorganic and Bioinorganic Chemistry*; Scott, R. A., Ed.; Wiley, 2011.

31. Veryazov, V.; Malmqvist, P. Å.; Roos, B. O. How to Select Active Space for Multiconfigurational Quantum Chemistry? *Int. J. Quantum Chem.* **111**, 3329–3338, (2011).

32. Heß, B. A.; Marian, C. M.; Wahlgren, U.; Gropen, O. A Mean-Field Spin-Orbit Method Applicable to Correlated Wavefunctions. *Chem. Phys. Lett.* **251**, 365–371, (1996).
